# Gas Permeability of the Anisotropic Structure of a Frame Made of Concrete with the Addition of a Biocomponent—Application in Livestock Buildings

**DOI:** 10.3390/ma19112257

**Published:** 2026-05-26

**Authors:** Elżbieta Janowska-Renkas, Dariusz Fabianowski, Igor Klementowski, Kinga Borek, Adam Koniuszy, Grzegorz Wałowski

**Affiliations:** 1 Department of Building Materials Engineering, Faculty of Civil Engineering and Architecture, Opole University of Technology, 48 Katowicka St., 45-061 Opole, Poland; e.janowska-renkas@po.edu.pl (E.J.-R.); d.fabianowski@po.edu.pl (D.F.); i.klementowski@po.edu.pl (I.K.); 2 Institute of Technology and Life Sciences, National Research Institute, Falenty, Al. Hrabska 3, 05-090 Raszyn, Poland; k.borek@itp.edu.pl; 3 Department of Renewable Energy Engineering, West Pomeranian University of Technology in Szczecin, 71-459 Szczecin, Poland; adam.koniuszy@zut.edu.pl; 4 Research Group on Energy Transformation and Industrial Systems, Research Centre for Innovative Technologies, Łukasiewicz Research Network—Institute of Artificial Intelligence and Cybersecurity, St. Leopolda 31, 40-189 Katowice, Poland

**Keywords:** water absorption, capillary action, ordinary concrete, lightweight concrete with added biocomponent, porous materials, gas permeability model, hierarchical decision structure, decision support system

## Abstract

The paper presents the results of experimental studies aimed at assessing thermal conductivity, compressive strength, water absorption and capillary action of samples in the form of ordinary concrete (reference sample—B1) and lightweight concrete with the addition of a biocomponent (C100) in the range of 3–31.2% porosity with varied morphology. Gas permeability studies were conducted for porous materials with an anisotropic structure. The measurement results indicate a significant effect of the type of material on thermal conductivity for B1, which is 3.05 W·(m·K)^−1^ and C100 equal to 0.09 W·(m·K)^−1^. On the other hand, the highest water absorption is demonstrated by C100, which is 99%, and the lowest by B1 equal to 2%. Tests were conducted for different gas permeability conditions using oxygen (O_2_), nitrogen (N_2_) and carbon dioxide (CO_2_). The basis for assessing gas permeability through porous beds is the gas flow resulting from the overpressure forcing this flow. The highest gas permeability coefficient at a flow resistance of 6 kPa for B1 was 2.7·10^−7^ m^2^, and for C100, 2.1·10^−7^ m^2^ at CO_2_ flow. The following issues were identified: scientific, identifying the lack of research on gas permeability testing for anisotropic concrete structures; application, identifying reports of premature failure of concrete structures in livestock buildings due to the effects of toxic substances. The novelty in the article is the indication of the gas permeability model and the performance of a comparative analysis (multi-criteria analysis) based on diagnostic features. In the hierarchical decision-making structure, gas permeability was used as one of the evaluation criteria, which can be assessed as a stimulant or destimulant depending on the climatic zone. The permeability of gas media is one of the features that allow for assessing the suitability of materials, among others, for small-sized prefabricated wall systems—the durability of both the element itself and any reinforcing inserts depends on permeability. The aim of this article was to compare the physical and functional properties of materials, such as thermal conductivity, water absorption, capillarity and gas permeability, in relation to the material composition. The research was of an application and engineering nature, focusing on macroscale functional parameters that are important from the point of view of the practical application of the tested building composites. The scientific problem is to indicate the lack of scientific research on the study of gas permeability in anisotropic concrete structures in livestock building conditions. The engineering use of hempcrete indicates its usefulness in various structural elements of livestock buildings.

## 1. Introduction

Buildings are subject to rigorous regulations specified in standards, both in terms of meeting the structural and mechanical parameters ensuring their durability, and in terms of ensuring the health and safety of people and animals living in them. This is determined by the quality of the air, which should be free from harmful pollutants, which is why air exchange in rooms intended for people to stay is so important.

Currently, the modification of the composition of concrete is closely linked to the management of industrial waste in accordance with the European Union directives on sustainable development and the reduction of the carbon footprint and the greenhouse effect. The search for materials as components of modified concrete is not limited to technological waste, but also to the management of waste from agricultural production using biocomponents as substitutes for aggregates or mineral additives (hemp shive, ash from the combustion of stems or plant husks).

The introduction of new components to concrete raises the question:

- How do manufactured composites, e.g., filling walls, affect the ventilation of rooms?

Unfortunately, the widespread use of gravity ventilation in rooms limits this process. This can cause health problems, including deterioration of cognitive abilities (increased drowsiness, impaired concentration) as well as the quality of environmental conditions (inadequate ventilation of rooms promotes the presence of various types of bacteria, viruses and allergens in the air). The negligence and errors of residents who treat air exchange in rooms as a source of heat loss mean that most houses and apartments do not meet the requirements of building law in terms of the minimum air exchange rate. This leads not only to poor air quality, but also to water vapor condensation and, consequently, increased moisture in the partitions. This, in turn, promotes the development of mold fungi, which adversely affect the immune system and cause allergies and various diseases among residents.

The production of building materials such as cement, steel, and ceramics requires huge amounts of energy. These processes are not only energy-intensive, but also emit significant amounts of greenhouse gases, contributing to global warming. Cement production, for example, is responsible for about 8% of global carbon dioxide (CO_2_) emissions [1]. Energy-intensive production processes, such as the firing of clinker at high temperatures, are major sources of these emissions. In addition, the extraction of raw materials and their transportation to factories also consume significant amounts of energy.

Hemp concrete, also known as “hempcrete”, is an eco-friendly building material that can provide a solution to the high energy consumption involved in the production of traditional building materials. In its original form, it consists of a mixture of hemp shives, lime, and water [2]. Porosity is one of the key properties of hempcrete, influencing its thermal and acoustic properties as well as the level of moisture absorption. The porosity of hempcrete results from both the naturally high porosity of the shives and the microstructures created by the binder hydration products.

Hempcrete is characterized by low thermal conductivity—Table 1, which makes it an excellent insulating material. The thermal conductivity of hempcrete is usually from 0.05 to 0.10 W/(m·K)^−1^, depending on the proportions of the components and the density of the mixture [3,4]. Traditional concrete, consisting mainly of cement, aggregate and water, has a much higher thermal conductivity compared to hempcrete. Typical thermal conductivity values (Table 1) of traditional concrete are from 1.4 to 3.2 W·(m·K)^−1^ [5,6]. High thermal conductivity makes traditional concrete ineffective as an insulating material [7].

Hempcrete, thanks to its low thermal conductivity, offers better insulating properties compared to traditional concrete. This makes it an attractive alternative to traditional building materials, especially in the context of energy-efficient and sustainable construction.

Porosity also translates into high water absorption of concrete, which is one of the key parameters influencing its durability and mechanical and physical properties. The ability of concrete to absorb and retain water has direct consequences for both the long-term durability of the material and its resistance to environmental factors such as frost, de-icing salts or chemicals. The process of water absorption in concrete depends mainly on the porous structure of the material, specifically on the size, distribution and number of capillary pores that are formed as a result of cement hydration and the proportions of the concrete mix [8,9].

Concrete water absorption is determined by two parameters:

(1) Total water absorption, which determines the maximum amount of water that concrete can absorb when exposed to direct contact with water for a long period of time;
(2) Capillary water absorption, which refers to the absorption of water through capillary pores under the influence of capillary forces, especially in conditions where concrete is in contact with moisture or groundwater [10]. The key role here is played by both open pores (which allow water to flow) and closed pores (in which water can accumulate but does not flow freely). The size of pores is also an important aspect—pores with a capillary diameter (from 10 nm to 10 µm) are responsible for most of the water transport in concrete [11]. Concrete water absorption depends on many factors, including the water/cement ratio, type of cement, admixtures and material curing conditions. One of the admixtures that seals the concrete matrix and reduces the water–cement ratio may be fluidized fly ash (FPL) [12].

The use of fly ash in concrete production has long been the subject of intensive scientific and technological research. Fly ash, as a by-product of coal combustion in power plants, poses an environmental problem related to its storage. One of the most promising solutions is its secondary use in construction, especially in concrete production. In recent years, special attention has been paid to FPL, which is produced in the process of coal combustion in a fluidized bed. FPL is characterized by different physicochemical properties compared to traditional fly ash, which creates new possibilities for its use in concrete technology [13,14]. FPL differs from conventional fly ash primarily in chemical composition and particle morphology. It contains higher amounts of reactive calcium and magnesium oxides and is characterized by a porous structure [15]. These properties mean that FPL can affect various aspects of concrete properties, such as strength, tightness, durability and the setting and hardening process [16]. The use of FPL in concrete can contribute to improving its mechanical and ecological properties. The use of FPL as a substitute for a portion of cement leads to a reduction in CO_2_ emissions associated with cement production, which is an important aspect of sustainable development [17,18]. Studies have shown that the addition of FPL to concrete can improve its compressive strength, frost resistance and resistance to aggressive chemicals [19,20]. One of the key areas of research concerns the effect of FPL on the rheological properties of the concrete mix. Due to its porosity and specific surface, FPL can change the amount of water needed to obtain the right consistency of the mix, which in turn affects the setting and hardening processes of concrete [21,22].

The durability of concrete with the addition of FPL is another important aspect of research. FPL can act like a pozzolan, reacting with calcium hydroxide formed during cement hydration, which leads to the formation of additional binding products, such as calcium silicate (CSH). This phenomenon can improve the tightness of the concrete structure by reducing its porosity and increasing its resistance to the penetration of aggressive substances such as chlorides and sulphates [23].

Theoretical considerations resulting from the interpretation of the hydrodynamics of gas flow through various porous media [24,25] are described by means of very diverse models [26], both mathematical and experimental, taking into account either rectilinear flow (as in the laminar model of Poissuille flow [27], or a more complex filtration process (according to the Darcy model [28], but also valid only for laminar flow), up to numerous modifications of these models for specific structural conditions of the bed, based on experimental criteria of fluid movement in closed spaces. The modifications known from the literature, such as those by Ergun [29], Carman [30], Forchheimer [31], and Windsperger [32], most often concern the designation flow resistance, although they are dedicated to granular media, or their special form in the form of filling column devices.

A major difficulty is in using the models proposed in the literature [26], as well as their adaptation to conditions other than those resulting from their assumptions—the process conditions are related to the very diverse structure of porous materials, especially in the context of the shape of pores, their cross-section, and mutual connections enabling fluid flow. And finally, the magnitude of porosity, the relatively high value of which does not always indicate a greater throughput of skeletonized porous materials.

The assessment of gas permeability through porous beds is important both for process and technological reasons. In both situations, numerous attempts are made to search for effective methods for predicting the permeability of porous materials, as well as effective ways of measuring and verifying the methods of this assessment [33]. The methods of measuring gas permeability through porous beds given in the literature are very diverse, and it can be assumed that the only common feature of these methods is the design of the samplers, although there are no clearly unified methods for this assessment in this respect. An additional difficulty in this respect is that the samples used for testing have different forms and shapes, and most often, they are model beds, which does not always correspond to real conditions. A separate aspect is that the assessment of gas permeability is usually carried out in one selected flow direction of the prepared sample, which, in relation to porous natural materials, leads to large quantitative distortions. This state of affairs does not facilitate the transfer of measurement results to real conditions, and does not facilitate the establishment of unambiguous criteria for transferring the scale. This leads to the individualization of methods for assessing gas permeability through porous beds, which are most often based on experimental formulas. The mechanisms resulting from the hydrodynamics of gas movement in porous beds are described in various ways; however, the basis for the assessment of the hydrodynamics are usually models based on the interpretation of Darcy’s law due to the different mechanisms [34] and forms of gas transport in porous beds (laminar or turbulent flow, diffusion, Knudsen transport, etc.), which in most cases occur simultaneously, making the correct interpretation of hydrodynamic processes a very complex problem, and one often difficult to describe unambiguously [34].

The assessment of process phenomena resulting from gas permeability may concern many technological aspects relating to the features (related to the properties) of such materials as concrete or concrete with the addition of biocomponents. These issues are the subject of the assessment of gas movement in natural conditions.

In the context of the gas permeability tests conducted in this work through porous materials, an attempt was made to link the results of these tests with process issues resulting from the technological premises of using porous materials. This connection was referred to the assessment of the technological quality of concrete with the addition of a biocomponent, related to its properties for construction technology. The basis for the assessment comprised the results of our own research, relating to the measurement of structural features of the tested porous deposits and gas permeability.

The aim of the research was to perform a comparative analysis of traditional concrete and hempcrete, thus demonstrating the differences in the characteristics of the tested materials in the context of the evaluation criteria for the hierarchical decision-making structure.

The following algorithm for the research scope was identified:

- Thermal conductivity, compressive strength, water absorption, capillary action;
- Gas permeability of the test materials depending on the gases: O_2_, N_2_, CO_2_ as a function of gas flow resistance relative to the gas flow rate;
- Implementation of the gas permeability coefficient model for the test materials;
- Comparative analysis for a hierarchical decision-making structure;
- Ecological solutions for livestock buildings using hempcrete.

## 2. Materials and Methods

The research material consisted of permanent skeletal structures:

(1) Traditional concrete—reference sample (B1) with porosity of 3%;
(2) Concrete with added biocomponent—hemp concrete—C100 (CS1) with porosity of 31.2% based on hemp shives on cement binder with the addition of fluidized fly ash (FPL) with the proportions of components presented in Table 2.

The hemp shives used in the study were shives from the industrial variety of Białobrzegi hemp with a thickness of 5–25 mm, which were cleaned of fibers and dust. They are widely used in construction as the main hemp component.

The binder in both materials was CEM I 42.5 R cement, which is characterized by high dynamics of early strength growth. It allows for shortening the production and storage time of prefabricated elements and limits or eliminates the costs of additional energy consumption in the maturation process. The composition, based on the main component in the form of Portland clinker in the amount of 95–100%, secondary components in the amount of 0–5% and a setting time regulator in the form of calcium sulphate, allows for the production of concrete at reduced temperatures. The study used a product from the Górażdże cement plant (Opole Province, Krapkowice, Poland).

The mineral additive used in the composition of the hemp component was FPL from the combustion of hard coal from the Łagisza Power Plant (Będzin, Poland) with a grain size predominantly <0.25 mm.

**Figure 1:**
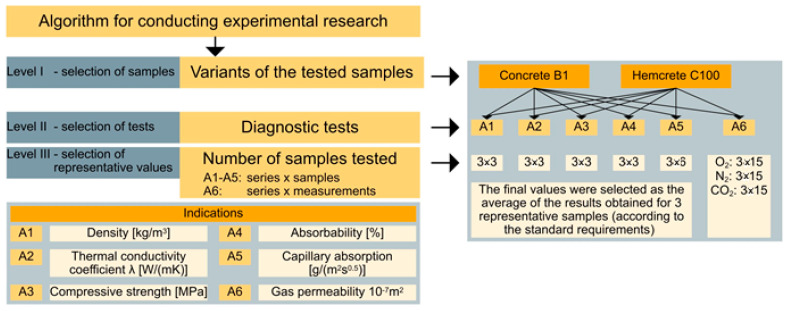
Block diagram of the experimental research program (prepared by D. Fabianowski).

The block diagram (Figure 1) shows the structure of the experimental research program conducted on two types of building materials: conventional concrete (Concrete—B1) and hempcrete (Hempcrete—C100). The aim of the experiment was to determine selected physical and mechanical parameters of both materials. A total of 54 samples were prepared and, depending on the standard requirements, for a given test method, 9 or 18 samples for each of the two concretes (conventional and hempcrete), respectively. For each type of test, three measurement series were performed, which ensured the repeatability of the results and enabled the assessment of the accuracy of the measurement methods. The high convergence of results in the triplicate series indicated good reproducibility of the research methodology. Therefore, in accordance with the guidelines included in the standards, representative samples were accepted for further statistical analyses and interpretation of results. For each of the test methods, a number of samples was taken into account, consistent with the minimum standard requirements, and in the case of series tests, the values were appropriately averaged. For example, for the compressive strength test, the final value was determined as the arithmetic mean of three samples, in accordance with the applicable material standards for this method.

Therefore, the following resultant number of samples for each test method was included in the test results presented in the article:

Thermal conductivity—3 samples;

Compressive strength—3 samples (average of 3 measurements);

Water absorption—3 samples;

Concrete density—3 samples;

Capillary rise—6 samples.

The use of these numbers of samples allowed for maintaining a balance between statistical reliability and compliance with standard requirements, while ensuring high accuracy and repeatability of the test results.

### 2.1. Experimental Stand—Thermal Conductivity, Compressive Strength, Water Absorption, Capillary Action

**Figure 2:**
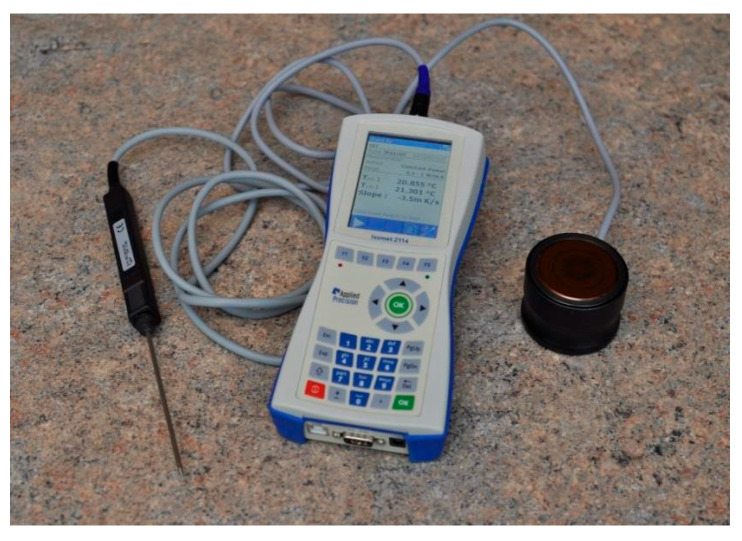
ISOMET 2114 heat conduction coefficient testing device (photography by I. Klementowski).

To measure the thermal conductivity coefficient, 3 samples of each material with dimensions of 100 mm × 100 mm × 100 mm were prepared. Sample conditioning consisted of storing all tested objects after 28 days of maturation in laboratory conditions in an atmosphere with controlled humidity and temperature (20 ± 1 °C, 50% RH ± 5%) for 72 h in accordance with ISO 12571:2013. The thermal conductivity coefficient (λ) was determined using the ISOMET 2114 apparatus (Figure 2) in accordance with ISO 8301:1991/Amd 1:2010. ISOMET 2114 uses the resistance method, which is consistent with the principles of the mentioned standard. The device can be used to determine thermal conductivity, volumetric heat flux and thermal diffusivity of cement-based composites, and the temperature measurement range is 15–50 °C with an accuracy of 1 × 10^−4^ W/(m·K). This test uses a surface probe with a measurement range of 0.04–0.3 W/(m·K). Before starting the tests, the device was calibrated on reference materials with known thermal properties. When starting the sample testing, the flattest and most uniform surface was selected to ensure even contact with the probe, and then a series of 3 measurements were performed by the device. The measured values for all tested objects were averaged to obtain the final result for a given material.

**Figure 3:**
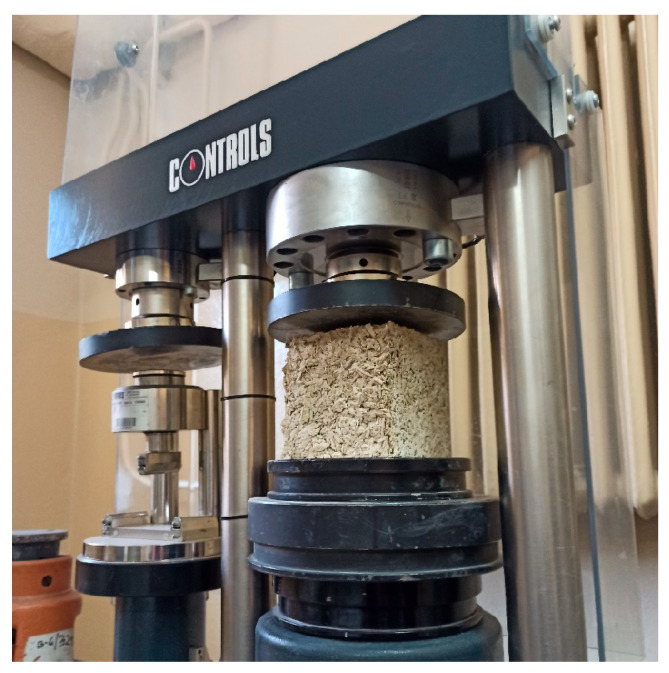
Pressure press—testing the compressive strength of the research material: concrete with biocomponent (C100) (photography by I. Klementowski).

**Figure 4:**
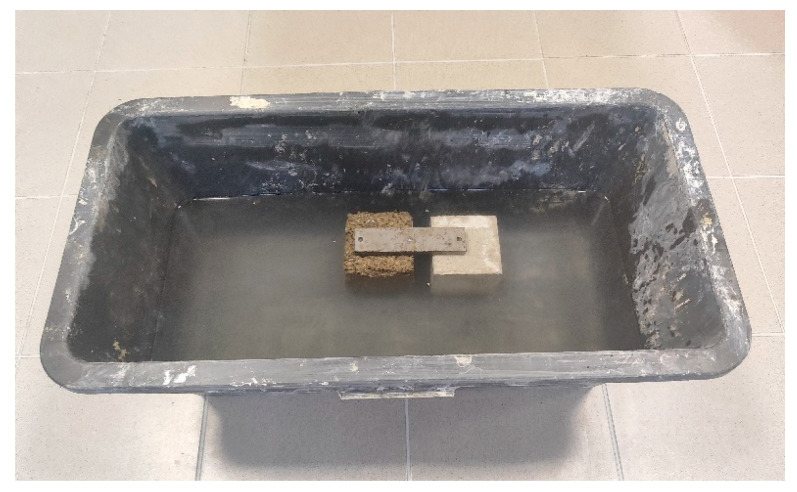
Water absorption test of research materials—from the left: concrete with biocomponent (C100), concrete (B1) (photography by I. Klementowski).

**Figure 5:**
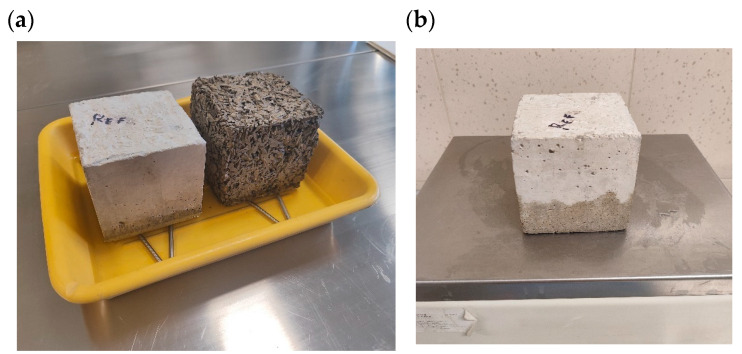
Capillary action (photography by I. Klementowski): (**a**) course of the capillary action test—from the left: concrete (B1); concrete with biocomponent (C100); (**b**) weighing of sample B1.

To better characterize the described materials, compressive strength values were also determined. The test was carried out in accordance with the PN-EN 12390-3 standard on 3 samples of each material (Figure 3):

The next step was to test the water absorption level in PN-88/B-06250 (Figure 4) and capillary action in PN-EN 772-11 (Figure 5) in accordance with the guidelines included in the standards.

Samples for water absorption testing were stored in laboratory conditions, similarly to those for strength testing, and tested after 28 days of curing. Samples were placed in a bathtub on 10 mm thick supports so that the base of the samples did not touch the bottom. Water at a temperature of 18 ± 2 °C was added to half the height of the samples. After 24 h, water was added to a level 10 mm above the height of the samples, and this level was maintained until the saturation was complete. Each day, the samples were removed, their surfaces wiped, and weighed to an accuracy of 0.2%. The process was continued until the difference between successive weighings was less than 0.2%. After complete saturation, the samples were dried at 105–110 °C until a constant mass was reached. Concrete water absorption, expressed as a percentage, describes the ratio of the mass of water absorbed by the sample to the mass of the sample in a dry state.

The capillary action test was conducted in accordance with the PN-EN 772-11 standard. For this purpose, 6 samples of each material were used. After 28 days of curing, the samples were dried to a constant mass at a temperature of 105–110 °C. Then, after cooling, all of the samples were immersed in water to a depth of 5 ± 1 mm for 24 h. During the experiment, the water level in the cuvettes was regularly checked and successively replenished. The samples were taken out, their surfaces wiped, and weighed to an accuracy of 0.1% of their total mass. Then, the water absorption coefficient of the tested materials was determined based on the averaging of the capillary absorption results for each sample with an accuracy of 1 g/(m^2^·s^0.5^).

### 2.2. Experimental Stand—Gas Permeability

**Figure 6:**
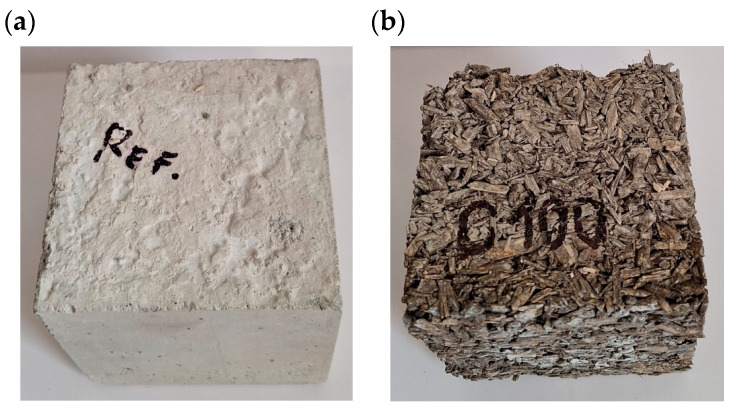
Sample of the research material (photography by G. Wałowski)—porous material with dimensions of 100 mm × 100 mm × 100 mm: (**a**) concrete, reference sample (B1), 3% porosity; (**b**) concrete with biocomponent (C100), 31.2% porosity.

Detailed experimental studies were carried out to assess the gas permeability of porous materials (cube shape) with varied structure and simultaneously varied process characteristics. The reference material sample was concrete (B1)—Figure 6a, and for comparative purposes, a combination of concrete with the addition of a biocomponent (C100) was used—Figure 6b.

**Figure 7:**
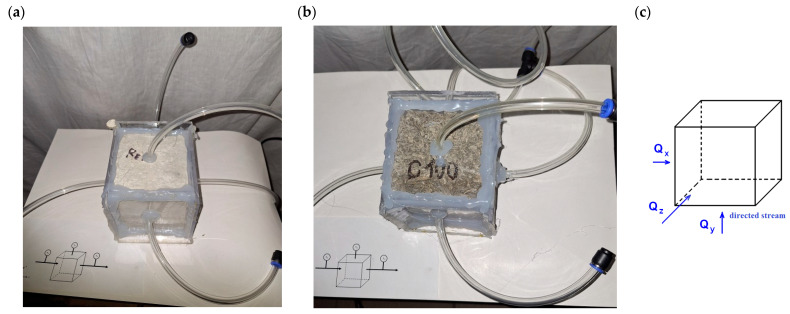
Measuring cell—flow channels for the tested sample (photography by G.Wałowski)—example flow in direction X: (**a**) concrete, reference sample (B1); (**b**) concrete with added biocomponent (C100); (**c**) gas flow diagram through the sample [own study—G.Wałowski]: Q_x_—gas flow in direction X, Q_y_—gas flow in direction Y, Q_z_—gas flow in direction Z.

The tests of samples (dimensions 100 mm × 100 mm × 100 mm) were carried out on a specially made measuring cell (Figure 7), the main elements of which are flow channels in which a sample of porous material is placed: concrete (B1)—Figure 7a, concrete with added biocomponent (C100)—Figure 7b. On the other hand, the gas flow could be directed with respect to an arbitrarily selected axis X, Y, Z for the measuring cell according to the scheme—Figure 7c. The flow directions were determined (according to the Cartesian method) in a conventional way, i.e., the X axis is the horizontal flow direction (horizontal plane), the Y axis is the vertical flow direction (vertical plane), and the Z axis is the frontal-horizontal flow direction (horizontal plane).

**Figure 8:**
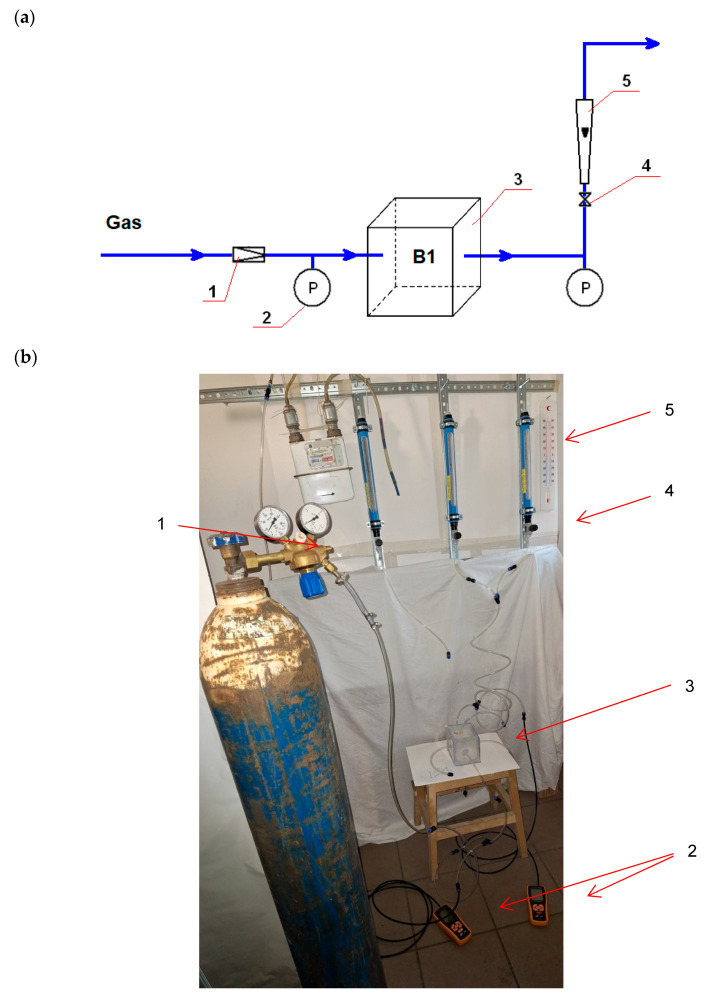
Test stand (photography by G.Wałowski): (**a**) gas flow diagram (oxygen): permeability through concrete (B1) in direction X, 1—pressure reducer, 2—digital manometer (P), 3—measuring cell: 100 mm × 100 mm × 100 mm, 4—valve, 5—rotameter (gas flow measurement); (**b**) example: concrete, reference sample (B1) using oxygen—view.

The permeability test stand for samples configured to a cubic shape (Figure 8) comprised a diagram (Figure 8a) and a system (Figure 8b) of the flow channel structure, which enables the measurement of gas permeability for each of the three main flow directions X, Y, Z, by plugging the cubic sample channel in the selected plane of the measuring cell (housing of the tested material)—Figure 7.

According to the schematic diagram (Figure 8a), the gas flowed through the test material depending on the pressure increase on the reducer. In this way, the gas permeability of the porous material was tested in a given flow direction, for example X.

For permeability tests, a gas was used as a working medium, which flowed through the test material (example of a test stand for a porous bed—Figure 8) in the pressure-free regime at a reference pressure on the reduction valve in the range of

- O_2_ (0.47–8.9) kPa, N_2_ (0.4–9.9) kPa, CO_2_ (0.34–6.9) kPa for concrete, reference sample (B1),
- O_2_ (0.49–8.61) kPa, N_2_ (0.47–9.8) kPa, CO_2_ (0.5–9.89) kPa for concrete with the biocomponent (C100).

Gas permeability tests were performed using rotameters and manometers for each test material, taking measurements in three series in each flow direction X, Y, Z (statistically not exceeding 20 measurements per series). To measure the pressure drop in a given measurement system (Figure 8), digital manometers installed in the flow measurement system before the gas inlet to the material sample were used. A battery of float rotameters was used to measure the gas flow. For the gases used: O_2_, N_2_, CO_2_, an appropriate scale conversion was applied with respect to the correction factor, taking into account the change in the parameters of the equation of state. By regulating the gas flow with valves for each system, the permeability characteristics of the tested samples were determined. All gas flow measurement results were referred to standard conditions (12 °C, 1013 hPa).

### 2.3. Research Scope and Methodology

As part of the basic research, the following were assessed:—thermal conductivity, compressive strength, water absorption and capillary action.

The basis for assessing the hydrodynamics of gas flow through the research materials is the characteristic of their permeability, which results from the pressure forcing this flow through the samples (research materials—Figure 6). The measure of permeability was the gas volume flow resulting from the available difference in pressure forcing the gas flow along a given axis: X, Y, Z on a porous material sample.

In order to learn the conditions for gas permeability through porous materials, detailed experimental studies were carried out in the scope of assessing flow resistance and the characteristic gas permeability coefficient for materials with a diversified structure.

An assessment of the permeability function and pressure drop on the porous bed was made, assuming the so-called directed flow X, Y, Z, characteristic for a cubic sample (Figure 7c). Based on the evaluation of the permeability function, the gas permeability coefficient was presented, comparing the research materials depending on the gases used—indicating the calculation models.

The measured technical parameters and material costs were used to conduct a decision analysis allowing for a comprehensive comparison of both tested samples.

## 3. Results

### 3.1. Thermal Conductivity, Compressive Strength, Capillary Action

The conducted tests showed significant differences in the conduction of thermal energy by selected materials (Table 3).

The thermal conductivity coefficient (λ) of hempcrete (C100) is significantly lower compared to traditional concrete (B1). The average value for C100 is approximately 0.0842 W·(m·K)^−1^, while for B1, it is 3.0480 W·(m·K)^−1^. C100 is characterized by very good insulating properties, which reduce the energy consumption for heating and cooling buildings, so it is beneficial from both an economic and ecological point of view. Thanks to the use of an appropriate partition thickness, C100 can meet the thermal conductivity requirements for the external building partition resulting from the technical conditions without the need for additional insulation in the form of, e.g., mineral wool or polystyrene.

The compressive strength of traditional concrete (B1) is 44.60 MPa—Table 4. This is a typical value for concrete used in structural construction, which must carry heavy loads.

C100 material is characterized by low strength (0.36 MPa), which is insufficient for use in structural building components. C100 can be used as a thermal insulation material, lightweight filler, or decorative element.

Due to the high content of organic material fragments, hempcrete is characterized by high porosity of the concrete matrix. This undoubtedly translates into low compressive strength and high potential for moisture accumulation in its structure. The tested water absorption of the C100 hempcrete sample was 99% (Table 5). For comparison, traditional B1 concrete, characterized by a compact structure, reached a value of 2%. Due to its high water absorption, the use of C100 concrete for external partition elements requires protection against atmospheric factors and water vapor diffusion.

Capillary absorption is the process of water moving up a porous material as a result of capillary forces. In the context of building materials such as concrete, it is of significant importance, affecting the durability of the structure, corrosion resistance of the reinforcement and resistance to freeze–thaw cycles. In traditional concrete, the intensity of this phenomenon depends mainly on the porous structure and the distribution of pores formed during cement hydration. In hempcrete, capillarity is partially limited by higher vapor permeability and specific microstructure, but the presence of shives increases the absorption and ability to absorb moisture. As a result, cement concrete is characterized by lower vapor permeability and uniform capillarity, but with long-term moisture retention, it promotes corrosion of the reinforcement. Hempcrete, thanks to its greater porosity and moisture regulation ability, improves the internal microclimate but requires protection against excessive soaking. The addition of hemp shives changes the nature of the capillarity of concrete, giving it pro-ecological properties, but it is necessary to design it appropriately to ensure durability and limit water absorption.

The capillary rise test again showed a huge difference between the matrix structure in hempcrete (C100) and that in traditional concrete (B1). Sample B1 achieved a better result of 0.01 g·(m^2^·s^0.5^)^−1^, showing high resistance to capillary rise. Sample C100 achieved an average value of 0.09 g·(m^2^·s^0.5^)^−1^—Table 6.

Microstructural analysis using SEM was not included in the scope of the current work for the following reasons:

- The study focused on the effect of composition on performance parameters, not on microstructure characterization,
- SEM requires separate samples, preparation and laboratory equipment that was not included in the planned research resources at the time of the project.

At the same time, the authors of the work are aware of the value of SEM analysis, especially in the context of interpreting gas permeability results and the formation of microporosity and cracks. Therefore, further research is planned, which will include the characterization of microstructure using SEM, in relation to composition variability and mechanical and transport properties.

### 3.2. Permeability of Porous Deposits

**Figure 9:**
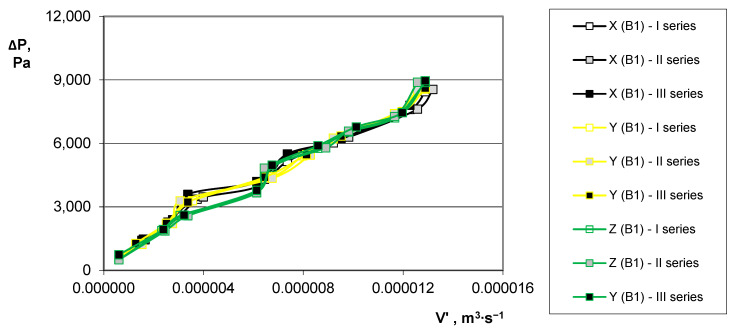
Permeability of the test material: concrete (B1), working medium: O_2_, cubic sample 100 mm × 100 mm × 100 mm (prepared by G. Wałowski).

The test results for O_2_ flow in the range of the volume flow rate V′ (0.6·10^−7^–13.2·10^−6^ m^3^·s^−1^) presented in Figure 9 show flow resistance ΔP in the range 0.5–8.9 kPa in relation to concrete, reference sample (B1)—Figure 6a, and the nature of changes in the gas permeability function is varied. In relation to the pressure drop on the porous bed, the measurement results indicate that practically for each flow direction X, Y, Z, the gas flow resistance increases with the increasing gas volume flow rate. This suggests a completely free gas flow, the flow rate of which results only from the permeability character of the given bed and is not related to the throttling of the medium. Analyzing the distribution of the curves, one obtains interpenetrating permeability characteristics, and their mutual deviation clearly depends on the working medium—O_2_, against the background of the structure of the porous material. The distribution of experimental points shows that the permeability of B1 significantly depends on the directionality of the gas flow. This indicates the effect of asymmetry of the flow with respect to the selected flow direction (axis) and, consequently, the anisotropic structure of this type of material. Moreover, these characteristics have a parabolic course, which indicates their similarity to the hydrodynamics of flow through closed channels. On the other hand, the nonlinear tendency of these characteristics indicates the dominance of turbulent flow, which is also associated with a deviation from Darcy’s law [35].

**Figure 10:**
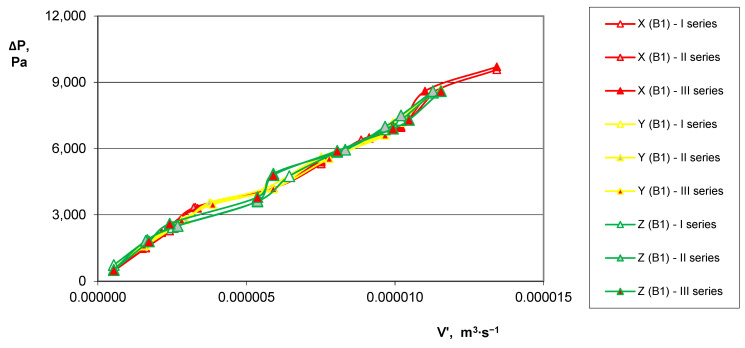
Permeability of the research material: concrete (B1), working medium: N_2_, cubic sample 100 mm × 100 mm × 100 mm (prepared by G. Wałowski).

The test results for N_2_ flow in the range of the volume flow rate (0.5·10^−6^–13·10^−6^ m^3^·s^−1^) presented in Figure 10 show flow resistance in the range 0.5–9.7 kPa in relation to B1—Figure 6a, and the nature of changes in the gas permeability function is varied. In relation to the pressure drop on the porous bed, the measurement results indicate characteristics and permeability properties similar to those for the oxygen flow.

**Figure 11:**
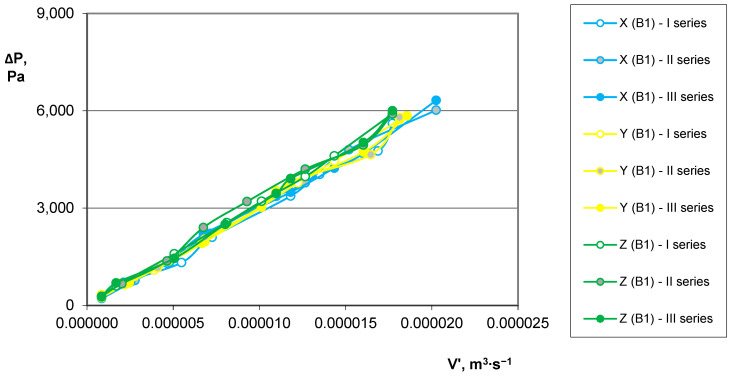
Permeability of the research material: concrete (B1), working medium: CO_2_, cubic sample 100 mm× 100 mm × 100 mm (prepared by G. Wałowski).

The research results presented in Figure 11 for CO_2_ flow in the volume flow range 0.8·10^−6^–20·10^−6^ m^3^·s^−1^ show flow resistance in the range of 0.2–6.3 kPa in relation to B1—Figure 6a, and the nature of changes in the gas permeability function is varied. In relation to the pressure drop on the porous bed, the measurement results indicate similar characteristics, but different permeability properties, compared to those for O_2_ and N_2_.

Comparing the research results presented in Figure 9 and Figure 10 in relation to Figure 11—the greatest flow resistance is shown by B1, through which O_2_ or N_2_ flows. On the other hand, B1, through which CO_2_ flows, has significantly lower flow resistance—this means that CO_2_ causes a reduction in the flow through B1 by up to 50%. Example: at a volume flow rate of 1·10^−5^ m^3^·s^−1^, the flow resistance is 6 kPa for the gas group (O_2_ or N_2_), and for CO_2_, the flow resistance is 3 kPa. Additionally, at constant flow resistance (6 kPa), the volume flow rate of CO_2_ is twice as large (2·10^−5^ m^3^·s^−1^) in relation to the gas group (O_2_ or N_2_), showing a value of 1·10^−5^ m^3^·s^−1^.

**Figure 12:**
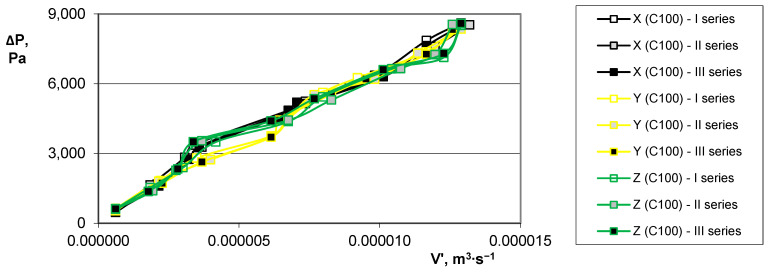
Permeability of the test material: concrete with added biocomponent (C100), working medium: O_2_, cubic sample 100 mm × 100 mm × 100 mm (prepared by G. Wałowski).

The research results for O_2_ flow in the range of the volume flow rate (0.6·10^−7^–132·10^−7^ m^3^·s^−1^) presented in Figure 12 show flow resistance in the range 0.6–8.5 kPa in relation to the concrete with added biocomponent (C100)—Figure 6b. The nature of changes in the gas permeability function is interpreted as in Figure 9, i.e., it is diversified, the gas flow resistance increases with the increasing gas volume flow rate, i.e., there is a completely free gas flow, the flow rate of which results only from the nature of the permeability of the given bed and is not related to the throttling of the medium.

**Figure 13:**
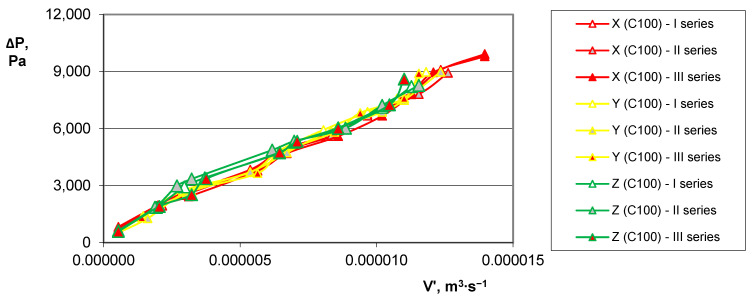
Permeability of the test material: concrete with added biocomponent (C100), working medium: N_2_, cubic sample 100 mm × 100 mm × 100 mm (prepared by G. Wałowski).

The test results for N_2_ flow in the range of the volume flow rate (0.5·10^−6^–14·10^−6^ m^3^·s^−1^) presented in Figure 13 show flow resistance in the range 0.6–9.8)kPa in relation to C100—Figure 6b. The nature of changes in the gas permeability function is interpreted as in Figure 10, i.e., it is diversified, and the characteristics and permeability properties occur analogously to those for the O_2_ flow.

**Figure 14:**
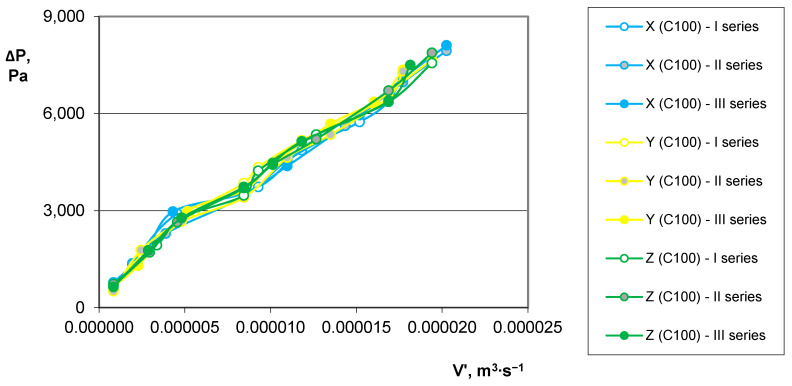
Permeability of the test material: concrete with added biocomponent (C100), working medium: CO_2_, cubic sample 100 mm × 100 mm × 100 mm (prepared by G. Wałowski).

The only difference in the permeability function occurs in the test results presented in Figure 14 for CO_2_ flow in the range of the volume flow rate (0.8·10^−6^–20·10^−6^ m^3^·s^−1^) in relation to C100—Figure 6b.

Taking into account Figure 9 and Figure 10, there is a similarity to the permeability in Figure 12 and Figure 13 in relation to the pressure drop on the porous bed—the measurement results indicate similar characteristics, but different permeability properties as for O_2_ and N_2_.

Comparing the test results presented in Figure 12 and Figure 13 in relation to Figure 14—the greatest flow resistance is shown by the concrete with added biocomponent (C100), through which O_2_ or N_2_ flows. On the other hand, C100, through which CO_2_ flows, shows significantly lower flow resistance—this means that CO_2_ causes a reduction of the flow through B1 by up to 25%. Example:—at a volume flow rate of 1·10^−5^ m^3^·s^−1^, the flow resistance is 6 kPa for the gas group (O_2_ or N_2_), and for CO_2_, the flow resistance is 4.5 kPa. Additionally, at constant flow resistance (9 kPa), the volume flow rate of CO_2_ is 1.6 times larger (2·10^−5^ m^3^·s^−1^) in relation to the gas group (O_2_ or N_2_), showing a value of 1.25·10^−5^ m^3^·s^−1^.

Another key issue is the comparison of Figure 11 and Figure 12, where a 30% increase in CO_2_ flow resistance was observed for the permeability function of C100 in relation to the permeability function of B1. It follows from this interpretation that CO_2_ is choked by 25% on the volume flow rate in relation to B1.

### 3.3. Gas Permeability Coefficient Model

The hydrodynamic parameters are known (gas flow, pressure drop across the bed, bed porosity and gas type); this is the value of the author’s permeability coefficient (1) defined in this way, which can be determined experimentally. Then:

(1) $$K_{V}=\frac{Q_{g}}{\sqrt{\frac{{\Delta P}_{exp}}{\rho_{g}}}}$$

where: *K_V_*—permeability coefficient, m^2^; *Q_g_*—gas stream, m^3^/s; Δ*P_exp_*– measured pressure drop, Pa; *ρ_g_*—density of gas, kg/m^3^.

If we relate the permeability coefficient defined in this way to the multi-plane flow direction X, Y, Z, (Q_x_, Q_y_, Q_Zz_) for each of them, we will obtain the following ((2)–(4)):

(2) $$K_{x}=\frac{Q_{x}}{\sqrt{\frac{{\Delta P}_{zm}}{\rho_{g}}}},$$

(3) $$K_{y}=\frac{Q_{y}}{\sqrt{\frac{{\Delta P}_{zm}}{\rho_{g}}}},$$

(4) $$K_{z}=\frac{Q_{z}}{\sqrt{\frac{{\Delta P}_{zm}}{\rho_{g}}}}.$$

The resulting geometric mean value of the permeability coefficient can be calculated as the root mean square (5):

(5) $$K_{V}=\sqrt{\frac{K_{x}^{2}+K_{y}^{2}+K_{z}^{2}}{3}}$$

where: *K_V_*—permeability coefficient, m^2^; *K_x_*—permeability coefficient for X—direction, m^2^; *K_y_*—permeability coefficient for Y—direction, m^2^; *K_z_*—permeability coefficient for Z—direction, m^2^.

In contrast to some models [36], this formula does not take into account the effect of gas viscosity on permeability, but it is an experimental quantity, which means that it includes all the properties of the gas that result from both temperature and pressure changes.

In reservoir engineering and materials research, the geometric mean is the standard for calculating average permeability because it best reflects the nature of flow in highly variable (heterogeneous) systems. It neutralizes extremes—single, very high values (e.g., a fracture in a sample) do not artificially inflate the average for the entire volume. It is consistent with nature—the distribution of permeability in rocks is usually log-normal, which means the geometric mean best represents the realistic potential for gas flow through a porous medium.

In the case of the gas permeability coefficient, the arithmetic mean is primarily used in a specific physical scenario, when material or bed layers are aligned parallel to the gas flow direction. Parallel flow—represents a situation in which gas can flow independently through each layer (layers with the highest permeability dominate the result). Homogeneous materials—if the samples are nearly identical, the difference between the arithmetic and geometric mean will be negligible. In most engineering studies, the arithmetic mean is considered an optimistic (overestimated) result. If the data come from a highly volatile material, the arithmetic mean may not reflect the actual resistance of the medium.

In the case of gas permeability, the harmonic mean is used when gas flows through layers arranged perpendicularly (in series) to the flow direction. Bottleneck—the harmonic mean is always the lowest of all the averages. It reflects the fact that in a series arrangement, the layer with the lowest permeability (the tightest) determines the flow rate through the entire material. Application—used in flow analysis through contaminated wellbore zones (so-called skin effect) or multilayer membranes, where the gas must pass through each segment sequentially.

**Figure 15:**
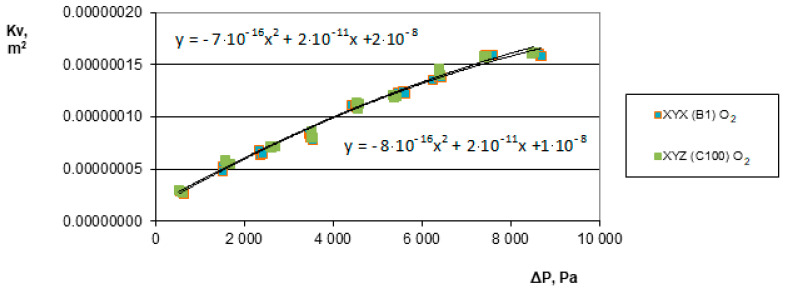
Gas permeability coefficient of research materials: concrete (B1) and concrete with added biocomponent (C100), working medium: O_2_, cubic sample 100 mm × 100 mm × 100 mm (prepared by G. Wałowski).

**Figure 16:**
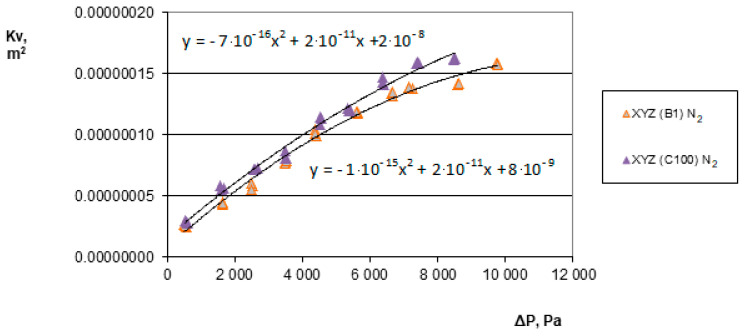
Gas permeability coefficient of research materials concrete (B1) and concrete with added biocomponent (C100), working medium: N_2_, cubic sample 100 mm × 100 mm × 100 mm (prepared by G. Wałowski).

**Figure 17:**
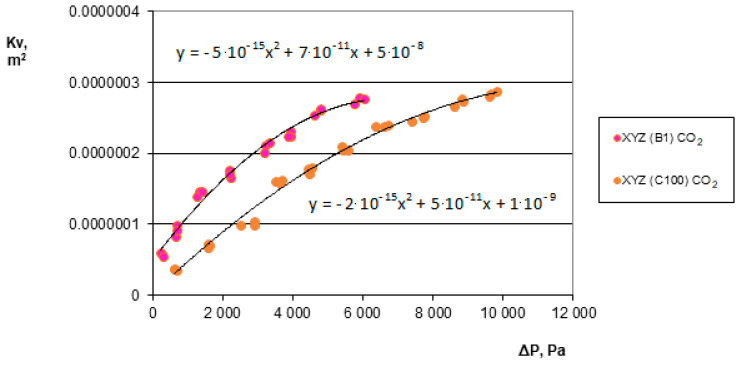
Gas permeability coefficient of research materials: concrete (B1) and concrete with added biocomponent (C100), working medium: CO_2_, cubic sample 100 × 100 × 100 mm (prepared by G. Wałowski).

The results obtained from the experimental determination of the permeability coefficient are presented in Figure 15, Figure 16 and Figure 17, which show the change in the value of this coefficient for a cubic sample of concrete (B1) and a concrete sample with added biocomponent (C100) depending on O_2_, N_2_, CO_2_—in relation to the directed flow. One can see the varied intensity of the gas flow in relation to the averaged XYZ directions, which indicates the strong anisotropy of this type of material.

The test results for the gas permeability coefficient using O_2_ for B1 and C100 presented in Figure 15 indicate the same function, taking into account the geometric mean for the XYZ direction.

The research results for the gas permeability coefficient using N_2_ for B1 and C100 presented in Figure 16 indicate that the higher gas permeability coefficient is for C100, taking into account the geometric mean for the XYZ direction.

The test results for the gas permeability coefficient using CO_2_ for B1 and C100 presented in Figure 17 indicate that the higher gas permeability coefficient is for B1, taking into account the geometric mean for the XYZ direction.

Taking into account the example measurement of flow resistance for 6 kPa, presented in Figure 15, Figure 16 and Figure 17, the test results indicate the highest gas permeability coefficient Kv in the following order: B1 for CO_2_ is 2.7·10^−7^ m^2^, C100 for CO_2_ is 2.1·10^−7^ m^2^, C100 for N_2_ is 1.3·10^−7^ m^2^, B1 for O_2_ is 1.3·10^−7^ m^2^, C100 for O_2_ is 1.3·10^−7^ m^2^, B1 for N_2_ is 1.2·10^−7^ m^2^. In the applied research materials, B1 (through which CO_2_ is passed) exhibits the Knudsen diffusion effect [37]. In these conditions, the velocity of particles on the walls of the porous bed is greater than zero, which increases the resultant gas flow. The gas permeability phenomenon indicates slippage called the Klinkenberg effect [38], i.e., free flow occurs at the molecular level. On the other hand, the B1 bed (through which N_2_ is passed) shows a lower gas permeability coefficient—there is no Klinkenberg effect.

A gas permeability model based on Darcy’s law describes the flow of gas through porous media (e.g., concrete, soil, ceramics) under laminar conditions. Darcy’s law assumes that the gas filtration rate is directly proportional to the pressure drop and inversely proportional to the gas viscosity and flow path length. For gases, their compressibility must be taken into account; therefore, the following parameters are used: volumetric flow rate, cross-sectional area through which the gas flows, dynamic viscosity of the gas, sample thickness (i.e., flow path length), system inlet and outlet pressure, and pressure at which the volume is measured (usually atmospheric pressure). The specific permeability coefficient, however, is a material property independent of the gas type.

The Klinkenberg effect (gas slippage) occurs in very small pores, where gas molecules “bounce” off the walls, artificially inflating the permeability result. This requires a correction for gases at low pressure. Considering the criterion Reynolds number, the model only works for laminar flows. If the gas flows too quickly (high pressure), turbulence occurs, and the Forchheimer equation must be used. The Klinkenberg effect is a key correction to Darcy’s law, applicable to gases flowing through media with very low porosity (e.g., high-performance concretes, shales, ceramics). In the classical Darcy model, the gas velocity at the pore wall itself is assumed to be 0 m·s^−1^. However, in small capillaries, the mean free path of gas molecules is comparable to the pore diameter. This results in so-called gas slippage—molecules “slide” along the walls, causing the measured flow to be greater than would be expected based on viscosity alone. Klinkenberg demonstrated that the measured gas permeability depends on the average gas pressure in the sample and is related to the liquid permeability (absolute permeability), which has the following characteristic properties: apparent gas permeability, Klinkenberg permeability (corresponding to the permeability of a liquid or gas at infinitely high pressure), average gas pressure in the sample, and Klinkenberg slip coefficient, which depends on pore geometry and the type of gas.

Prof. Wałowski’s original gas permeability model is an extension of the classical Darcy’s law for gases and takes the following into account: gas compressibility, the Klinkenberg effect (slip), and a more realistic description of flow in fine pores. The Wałowski model’s concept is characterized by permeability that is not constant, depends on pressure (due to the Klinkenberg effect), and takes into account changes in gas density. The model combines two key effects: the square of the pressure (gas compressibility) and the Klinkenberg correction (slip). It significantly better describes flow at low pressures and flow in very fine pores. The model is more accurate than pure Darcy’s law and simple gas models without slip correction. Physical interpretation of the model indicates that at high pressure, fluid-like behavior (Darcy) occurs, while at low pressure, slip becomes more important, resulting in greater flow. The Wałowski model is an extended Darcy’s law for gases, taking into account compressibility and the Klinkenberg effect, used in flow analysis under difficult conditions (low permeability, low pressure). The gas permeability coefficient in Wałowski’s approach is not treated as a constant (as in classical Darcy’s law), but as a pressure-dependent quantity.

### 3.4. Comparative Analysis of Materials Based on Results Obtained from Laboratory Tests

**Figure 18:**
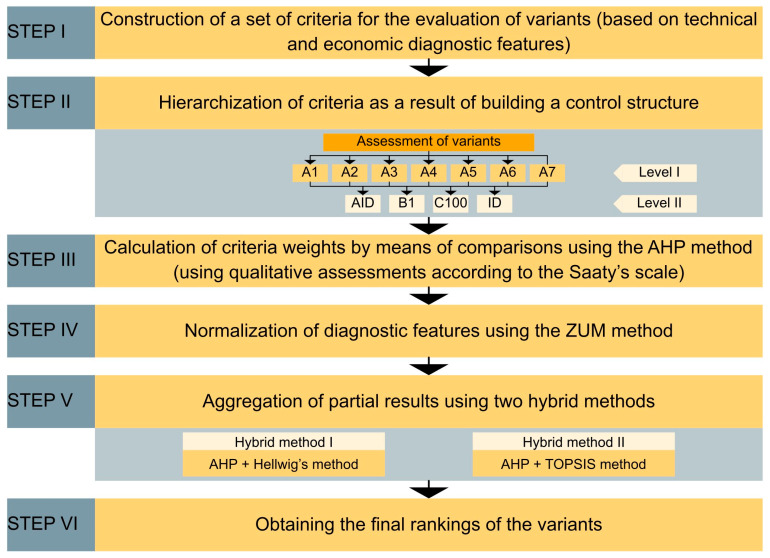
Graphical interpretation of the algorithm for assessing the tested variants B1 and C100—symbols are given in Table 7 (prepared by D. Fabianowski).

A graphical interpretation Decision Support System (DSS) of the evaluation process of the tested samples is given in Figure 18. The comparative analysis of the tested samples was carried out assuming their use as a filling material for external walls in frame buildings.

The analysis of the tested samples was carried out based on diagnostic features (evaluation criteria), which are technical parameters obtained from laboratory tests (Table 7) supplemented by the cost of materials used to make the samples (economic criterion). The criteria adopted in this way constitute quantitative diagnostic variables.

The hierarchical decision structure was built as a two-level structure. The first level of the hierarchical tree consists of criteria, the second of decision variants: reference concrete (B1), concrete with biocomponent (C100) and ideal (ID) and anti-ideal (AID) variants. The technical parameters of the reference variants were selected based on various materials used as structural elements of external partitions. Qualitative assessments were used in the process of determining the weights of the criteria. The weights of the criteria were determined using the Analytic Hierarchy Process (AHP) method by Saaty [39].

Pairwise comparisons were made using the classic 9-point scale [40]. Odd values 1–9 indicate equivalence, slight, strong, very strong and absolute advantage, respectively. Intermediate even values are used optionally. Diagnostic features are divided into two categories: stimulants (S) and destimulants (D) (designations in Table 7). The A6 gas permeability criterion can be assessed both as a stimulant and a destimulant, depending on, for example, the average annual outside temperature, climate zone or the type of external partition construction. For a moderate climate and the application of assumptions as for low energy or zero energy buildings equipped with recuperation systems that also take care of the appropriate air quality, uncontrolled gas flow associated with the loss of thermal energy will be an unfavorable phenomenon.

The standardization of dissimilar diagnostic features was carried out using the Zero Unitarization Method (ZUM). As the only standardization method, it meets all seven postulates set for standardization methods, which allows it to be defined as a universal method. In addition, its undoubted advantage is the transformation of diagnostic features to a convenient range of values in the analysis <0, 1>. The final evaluation of the variants was conducted using two hybrid methods: AHP + Hellwig’s taxonomic method and AHP + TOPSIS (Technique for Order Preference by Similarity to Ideal Solution)—both methods from the MCDA (Multiple-criteria decision analysis) group. Both methods allow for direct application of diagnostic features without the need to transform them to the internal scale of other methods, which causes loss of information. In the cases of both hybrid methods, the use of the aggregate procedure leads to obtaining a synthetic variable constituting a set of evaluations of individual material solutions (variants).

The AHP and TOPSIS methods are known and widely cited in the literature [39,40,41,42]; therefore, the presentation of their calculation algorithms is omitted, while the formulas normalizing the ZUM method are given below.

Formulas normalizing the ZUM method:

- Stimulant (6):

(6) $$z_{ij}=\frac{x_{ij}-\underset{i}{\min}\;x_{ij}}{\underset{i}{\max}\;x_{ij}-\underset{i}{\min}\;x_{ij}}$$

- Destimulant (7):

(7) $$z_{ij}=\frac{\underset{i}{\max}\;x_{ij}-x_{ij}}{\underset{i}{\max}\;x_{ij}-\underset{i}{\min}\;x_{ij}}$$

Hellwig’s taxonomic method:

- After normalization using UTI, a development pattern is assumed, by taking the maximum values of individual diagnostic variables (8):

(8) $$m_{0j}=\underset{i}{\max}\;z_{ij}$$

- For each of the variants tested, the distance from the development pattern is determined (9):

(9) $$d_{i}=\sqrt{\sum_{j=1}^{k}{\left(z_{ij}-m_{0j}\right)}^{2}}$$

- Calculation of the relative synthetic measure (10):

(10) $$m_{i}=1-\frac{d_{i}}{d_{0}}\;dla\;i=1,2,\ldots ,n$$

where:

(11) $$d_{0}=\bar{d}+2s_{d}$$

(12) $$\bar{d}=\frac{1}{n}\sum_{i=1}^{n}d_{i}$$

(13) $$s_{d}=\sqrt{\frac{1}{n}\sum_{i=1}^{n}{\left(d_{i}-\bar{d}\right)}^{2}}$$

**Figure 19:**
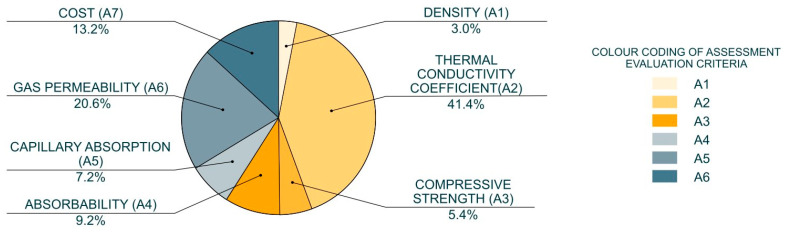
Graphic interpretation of the share of individual criteria in the final assessment (prepared by D. Fabianowski). Legend: A1 –density, A2—thermal conductivity coefficient, A3—compressive strength, A4—Absorbability, A5—capillary absorption, A6—gas permeability, A7—cost.

The results of the analysis are presented in Table 8, and the graphic interpretation of the share of individual criteria is presented in Figure 19. The highest weights were given to criteria related to thermal conductivity (A2) and gas permeability (A6). These are the parameters of the highest importance in the case of using the tested concretes for the external curtain walls of buildings.

**Figure 20:**
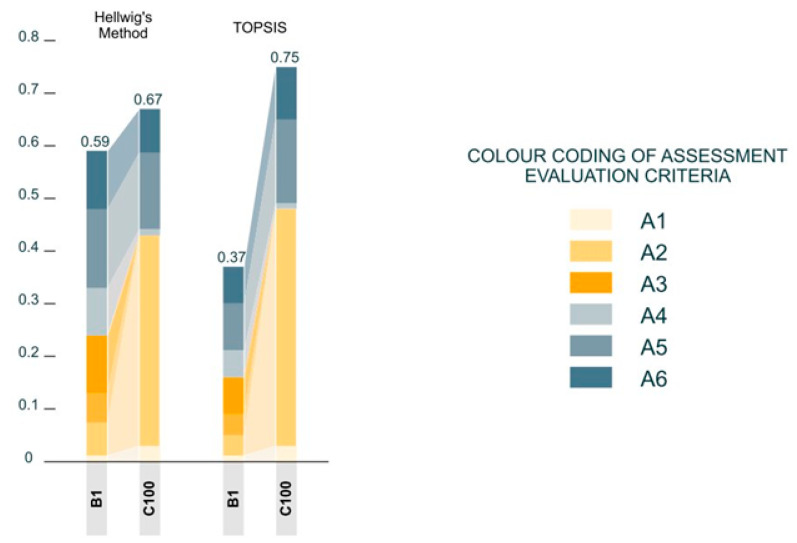
Values of partial assessments according to individual criteria in the collective assessment—on the vertical axis, a scale of unassigned grades was used in the range of values <0, 1> (prepared by D. Fabianowski). Legend: A1—density, A2—thermal conductivity coefficient, A3—compressive strength, A4—Absorbability, A5—capillary absorption, A6—gas permeability, A7—cost.

The use of the proposed methods allows for the ordering of the decision-making process and the possibility of standardizing and aggregating dissimilar diagnostic features leading to the selection of the optimal variant. The analysis results obtained as a result of the use of two variants of hybrid methods clearly indicate the advantage of concrete with the addition of a biocomponent (C100). The decisive influences on such an assessment were the A2 criterion and the very good insulation of C100. The values of partial assessments according to individual criteria in the collective assessment are presented in Figure 20. The differences in the assessments obtained using both methods result from different mathematical algorithms.

With the growing complexity of decision-making issues, the need to support the process with mathematical apparatus (DSS) increases. Its use leads to the ordering of the decision-making process and, as a result, to obtaining an optimal solution. The universal nature of decision-making algorithms allows them to be used in very different applications. In construction, these can be, for example, the optimization of material solutions (presented in the work), construction solutions (e.g., prefabrication systems), production technology, selection of machinery, method of investment implementation or several integrated issues simultaneously. Another promising application is the implementation of multi-criteria decision-making methods in MR&R (Maintenance, Repair, and Rehabilitation) issues. The challenge is to properly prioritize construction objects (e.g., buildings, bridges) based on the urgency of repair work. Prioritization is based on assessments of the technical condition of their components and is essential for the proper allocation of, often limited, financial resources and the planning of maintenance, repair, and rehabilitation (MR&R) activities. MCDA methods are used, among other things, to assign weights to individual structural elements based on their impact on the “lifespan” of a structure and to determine the interrelationships between elements based on their technical condition.

Decision-making algorithms are used at all stages of the life cycle of an object, starting from the concept through the design process, construction, maintenance and preservation of existing objects up to demolition. They are widely used not only in construction but also in related fields such as spatial planning and energy or environmental protection with consideration for sustainable development.

### 3.5. Hempcrete Livestock Buildings—An Ecological Solution for the Future

The construction sector has a greater impact on carbon dioxide emissions than all other sectors. Hempcrete livestock buildings are a modern approach to agricultural construction that is gaining increasing popularity. Hempcrete, also known as hemp concrete, is an ecological composite that combines the advantages of sustainable production with excellent performance properties, and that can replace cement-based products. It is made from a mixture of hemp shives, lime or another mineral binder and water. In the context of livestock buildings—such as barns, stables or pigsties—hempcrete stands out as an exceptionally functional material, adapted to contemporary ecological and technological requirements [43,44,45,46,47].

Hempcrete is an innovative building material that is gaining popularity due to its unique properties. In the context of livestock housing, its advantages are particularly important and affect the comfort and safety of animals, as well as the durability of the structure. Here is a detailed discussion of the most important features of hempcrete [44,45,46,47,48,49]:

- Thermal and acoustic insulation—The porous structure of hempcrete provides excellent thermal and acoustic insulation. As a result, there is a stable temperature inside the buildings, which is crucial for the proper welfare of animals.
- Humidity regulation—Hempcrete has the ability to “breathe”, which means that it can absorb excess moisture from the air and, if necessary, release it back, maintaining optimal conditions in the rooms. Stabilizing moisture levels prevents mold and fungi from forming, which can be particularly problematic in livestock facilities.
- Ecology—All of the ingredients in hempcrete are of natural and renewable origin. It consists of natural, renewable ingredients—hemp, lime and water. The production of hempcrete has a negative carbon footprint. After the building is no longer in use, the material is fully biodegradable (recyclable), which minimizes the impact on the environment. This means that hempcrete does not contribute to environmental pollution, making it an ideal choice for those looking for nature-friendly solutions.
- Resistance to mold and fungi—The natural antibacterial properties of hempcrete mean that it limits the growth of microorganisms. Additionally, its porous structure allows for rapid moisture removal, which significantly reduces the risk of mold and fungi, even in a humid environment. These properties are particularly beneficial in livestock buildings, where the presence of moisture can pose a major problem for animal health and the durability of the structure.
- Lightness—Hempcrete is lighter than traditional concrete, which makes it easier to transport, store and install. The lower weight of the material also means less load on the building structure, which can be important in the case of larger livestock facilities. Thanks to this property, hempcrete can be used in places where traditional materials would be too heavy or expensive to transport and install.

Limitations of hempcrete [50,51]:

- Low mechanical strength—hempcrete is not suitable for use as a heavy-duty structural element, because its compressive strength is significantly lower than that of traditional concrete. It requires additional load-bearing elements, such as wooden or steel frames, which can increase construction costs and complicate the design and construction process.
- High initial costs—although hempcrete is an ecological material, its use is associated with higher initial costs. These include, among others, specialist raw materials, limited availability of ready-made mixtures and the need to employ qualified specialists for its application. In addition, the construction process may require the use of non-standard technologies and tools, which also increases the investment costs.
- Longer drying time—hempcrete is characterized by a longer drying time compared to traditional building materials, which extends the investment implementation schedule. Depending on weather conditions and the thickness of the applied layer, the drying process can take from several weeks to several months. This requires careful planning of construction works and appropriate conditions for drying the material, which can be an additional challenge, especially in a climate with high humidity.
- Lack of standardization and availability of the material—There are currently no uniform norms and standards for the production and use of hempcrete. This can lead to a large variability of the material’s properties depending on the region and manufacturer, which is a significant barrier to its wide application. Additionally, in many places in the world, the availability of high-quality components is limited, which makes its implementation on a larger scale difficult.
- Need for specialist experts—The process of construction using hempcrete requires advanced technical knowledge and experience in working with composite materials. Improper preparation of the mix or incorrect application can negatively affect the properties of the final product. The lack of qualified specialists in this field can be a significant limitation, increasing investment costs and the risk of implementation errors.

**Figure 21:**
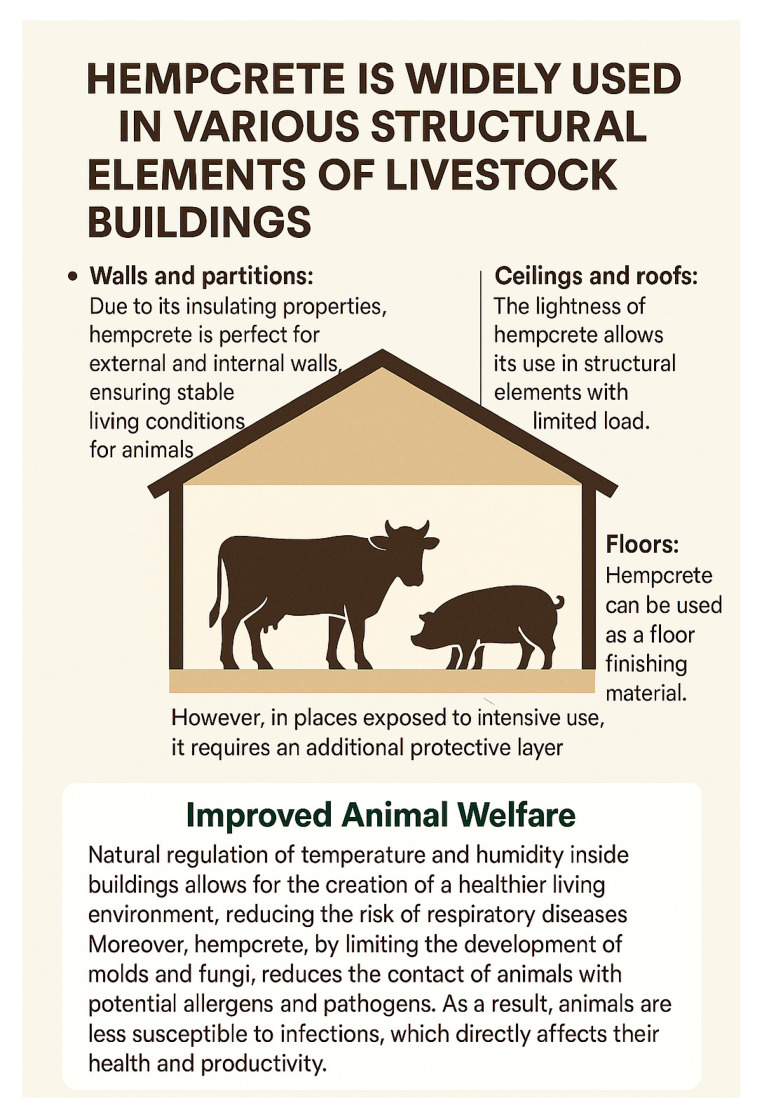
Hempcrete is widely used in various structural elements of livestock buildings [44,52,53] (prepared by K. Borek).

Hempcrete is widely used in various structural elements of livestock buildings [44,52,53]—Figure 21:

- Walls and partitions: Due to its insulating properties, hempcrete is perfect for external and internal walls, ensuring stable living conditions for animals.
- Ceilings and roofs: The lightness of hempcrete allows its use in structural elements with limited load.
- Floors: Hempcrete can be used as a floor finishing material. However, in places exposed to intensive use, it requires an additional protective layer.

The use of hempcrete in livestock buildings translates directly into improved animal welfare, due to the properties [43,44,45,46,47,49]. Natural regulation of temperature and humidity inside buildings allows for the creation of a healthier living environment, reducing the risk of respiratory diseases. Moreover, hempcrete, by limiting the development of molds and fungi, reduces the contact of animals with potential allergens and pathogens. As a result, animals are less susceptible to infections, which directly affects their health and productivity.

Building with hempcrete requires planning and experience. The process usually takes longer than with traditional building materials because the mixture must dry and harden before it reaches full strength. It is worth noting, however, that while the initial costs may be higher, the lower operating costs and environmental benefits make hempcrete a viable choice in the long run [53].

Although hempcrete offers many advantages, it is not without its limitations. The most important of these are as follows:

- Lower mechanical strength up to 1 year of use [45]. Compared to traditional concrete, hempcrete cannot be used in load-bearing elements, which limits its use.
- Need for experience [43]. Construction with hempcrete requires knowledge and skills, which can be a barrier for less experienced contractors.
- Sensitivity to moisture during construction [44]. During drying, hempcrete requires protection from excessive moisture, which can affect construction time and costs.

Hempcrete livestock buildings are a conscious choice for those looking for sustainable, ecological and animal-friendly solutions. Although this material is associated with some technical challenges, its advantages—including excellent insulation properties, natural moisture regulation and minimal environmental impact—make it the future of livestock construction [54].

Choosing hempcrete is an investment not only in modern technologies, but also in a healthier and more sustainable living environment for animals and people.

Numerous scientific studies confirm the properties of this material. Hygrothermal and mechanical properties of hempcrete depend on various factors: the binder-to-hemp ratio, the level of compaction, water content and the design of the mix. For example, increasing the share of porous hemp shive in the hempcrete mix reduces the dry density and the thermal conductivity. In addition, increasing water content in the mixtures tends to increase the thermal conductivity of hempcrete mixtures [44,51,55,56]. Jirgensone et al. [43] conducted hygrothermal monitoring in multilayer walls insulated with hempcrete, indicating no risk of mold and stability of thermal conditions. Its effectiveness in regulating humidity and thermal insulation, which is crucial in livestock buildings, was emphasized. Walker et al. [45] investigated the effect of binder type on mechanical strength and durability, including resistance to freezing and thawing, exposure to salts, and biodegradation. Hemp-lime concretes made with hydrated lime and pozzolanic binder (GGBS and metakaolin) were compared with concretes containing hydraulic lime and cement (commercial or local binders containing PC). It was shown that

- Lime-pozzolan binder is carbonated with a small amount of pozzolanic hydrates; a slight decrease in hydration products is noticeable, compared to commercial or PC binders. Lime-pozzolanic binders with water-retaining additives, on the other hand, show a slight increase in hydration products;
- Lime-pozzolanic concretes achieve strength similar to that of hydraulic lime and cement, after 1 year. This is because concretes containing hydraulic lime and cement have the highest compressive strength, but as the concrete ages, other factors, such as carbonation, affect the strength;
- Hemp-lime concrete achieved lower flexural strength, after 3 months and after 1 year, than concretes containing hydraulic lime and cement. However, water-retaining additives improved the flexural strength of lime-pozzolanic concrete;
- Water-retaining agents were found to improve the frost and thaw resistance of the lime-pozzolanic binder;
- After 1 month of exposure to a salt environment, no damage was observed in hemp-lime concrete. This concrete exhibits high ductility of pore walls, which withstand the expansive pressure of salt crystallization.
- Hemp-lime concrete is resistant to biodeterioration in environmental conditions similar to those found on a construction site.

Studies by Shea et al. [46] have shown that the use of hemp-lime concrete as insulation helps maintain comfortable summer conditions. De Bruijn and Johansson [55] showed that a higher amount of hemp in the mixtures resulted in better thermal properties, Abdellatef et al. [44] studies showed that the thermal and mechanical properties of concrete depend on its density. Additionally, a higher content of hemp shive in the mixtures can lead to the development of products suitable for use in infill walls. According to the studies, it was determined that new formulations of hydrated lime and crushed brick have comparable mechanical values to metakaolin and hydraulic lime. It was also indicated that water content is an important parameter due to its significant effect on the mechanical and thermal properties of hempcrete samples. A lower amount of water (≤2.5 kg) leads to worse mechanical properties of some samples due to incomplete hydration caused by the high suction ability of the hemp shive.

The authors pointed out that research on hempcrete is required, but despite technical challenges such as mechanical strength or the need for experience on the part of contractors, hempcrete stands out as a future material that promotes animal health and environmental protection.

## 4. Discussion

### 4.1. Study on Thermal Conductivity, Compressive Strength, Water Absorption, Capillary Action, and Gas Permeability of Ordinary Concrete (B1), Lightweight Concrete with a Biocomponent (C100), and Hempcrete with Varied Porosity and Morphology

This study compares the thermal conductivity, compressive strength, water absorption, capillary action, and gas permeability of ordinary concrete (B1), lightweight concrete with a biocomponent (C100), and hempcrete. The analysis focuses on different gas permeability conditions using oxygen, nitrogen, and carbon dioxide, including a comparative multicriteria analysis.

Thermal conductivity is a critical property that determines how well a material conducts heat. For ordinary concrete (B1), thermal conductivity typically ranges between 1.0 and 2.0 W/m·K, depending on its density and moisture content [57,58]. Lightweight concrete with a biocomponent (C100) generally exhibits lower thermal conductivity due to its higher porosity and the insulating properties of the biocomponent. Studies have shown that the thermal conductivity of such concretes can range from 0.3 to 0.8 W/m·K [58]. Hempcrete, which is a highly porous material made from hemp fibers and a lime-based binder, has even lower thermal conductivity, typically ranging from 0.04 to 0.1 W/m·K, making it an excellent insulating material [57,58].

Compressive strength is a measure of a material’s ability to withstand compressive forces. Ordinary concrete (B1) typically has a high compressive strength, ranging from 20 to 40 MPa, depending on the mix design and curing conditions [59]. Lightweight concrete with a biocomponent (C100) generally has lower compressive strength due to its higher porosity and the use of less dense aggregates. The compressive strength of such concretes typically ranges from 5 to 15 MPa [59]. Hempcrete, on the other hand, has a much lower compressive strength, typically ranging from 0.5 to 2.0 MPa, due to its high porosity and the weak bond between the hemp fibers and the binder [59,60].

**Figure 22:**
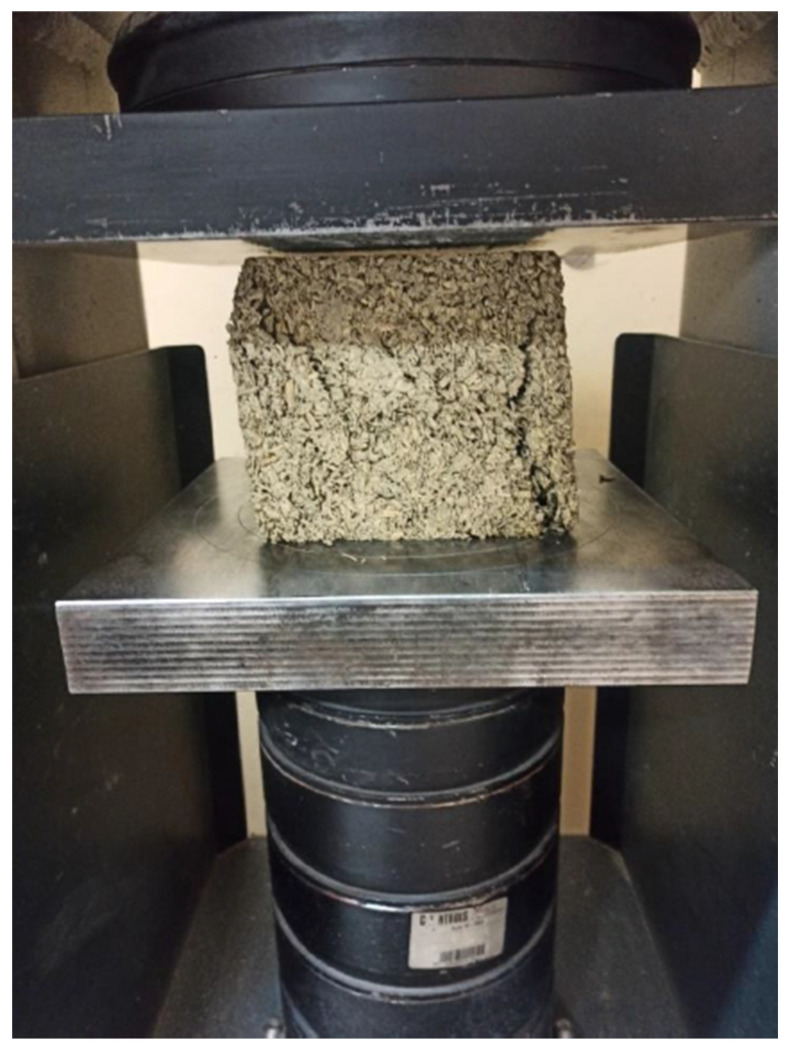
Hempcrete sample after compressive strength test (prepared by I. Klementowski).

An example from our own research indicates that after the compressive strength test, the analyzed hempcrete sample (Figure 22) retained overall geometric continuity, but its surface showed a distinct pattern of vertical and near-vertical cracks. The crack planes developed primarily from the lateral edges of the sample and converged towards its central part, indicating a concentration of damage in the central zone and a gradual opening of the material structure under the influence of transverse stresses induced by axial compression.

In the central part of the specimen, no continuous, large-width cracks or a distinct shear plane were observed. However, minor discontinuities, micro-cracks, and local zones of matrix weakness were visible. Their irregular course is a consequence of the heterogeneous structure of hempcrete, in which a stiff mineral matrix interacts with a lightweight, irregularly distributed organic filler. This failure pattern can be considered normal for a lightweight hemp–mineral composite. The pattern of cracks is essentially uniform on both sides of the specimen, and their direction corresponds to the typical splitting mechanism of a material subjected to compression. No unilateral crushing, significant sample tilt, or dominant oblique shear plane spanning the entire height of the element was observed, indicating correct, axial load application. Compared to plain concrete, the failure was less abrupt. In cement concrete, under compressive loading, distinct oblique shear planes, lateral cones of failure, or brittle failure of the specimen often develop. The tested hempcrete (Figure 22) is dominated by vertical cracks, whose progression is partially disrupted by the material’s heterogeneous structure, the presence of hemp shives, and the porous mineral matrix. The visible pattern of cracks, converging towards the center of the sample, indicates a gradual loss of load-bearing capacity due to the development of vertical cracks, local structural loosening, and partial material debonding at the edges. The similar pattern of failure observed in the remaining samples confirms the repeatability of the failure mechanism and indicates that the test procedure was correct for the analyzed hempcrete.

Water absorption and capillary action are important properties that influence the durability and moisture transport in concrete. Ordinary concrete (B1) generally has low water absorption due to its dense microstructure, with absorption rates typically ranging from 1% to 3% by weight [61]. Lightweight concrete with a biocomponent (C100) tends to have higher water absorption rates, typically ranging from 5% to 10% by weight, due to its higher porosity and the hydrophilic nature of the biocomponent [61]. Hempcrete exhibits very high water absorption rates, typically ranging from 10% to 20% by weight, due to its highly porous structure and the hydrophilic nature of the hemp fibers [61,62].

Gas permeability is a measure of how easily gases can pass through a material. Ordinary concrete (B1) generally has low gas permeability due to its dense microstructure, with intrinsic gas permeability coefficients typically ranging from 10^−19^ to 10^−17^ m^2^. Lightweight concrete with a biocomponent (C100) tends to have higher gas permeability due to its higher porosity, with intrinsic gas permeability coefficients typically ranging from 10^−17^ to 10^−15^ m^2^. Hempcrete exhibits very high gas permeability due to its highly porous structure, with intrinsic gas permeability coefficients typically ranging from 10^−15^ to 10^−13^ m^2^ [63,64].

Comparative multicriteria analysis—the following Table 9 provides a comparative analysis of the key properties of ordinary concrete (B1), lightweight concrete with a biocomponent (C100), and hempcrete [61,63,64].

The study highlights the significant differences in thermal conductivity, compressive strength, water absorption, capillary action, and gas permeability between ordinary concrete (B1), lightweight concrete with a biocomponent (C100), and hempcrete. Ordinary concrete exhibits high compressive strength and low thermal conductivity, making it suitable for structural applications. Lightweight concrete with a biocomponent offers a balance between thermal insulation and mechanical strength, making it suitable for lightweight structural applications. Hempcrete, with its extremely low thermal conductivity and high gas permeability, is ideal for insulation and non-structural applications where high moisture transport is acceptable. The choice of material depends on the specific requirements of the application, including mechanical strength, thermal insulation, durability, and gas permeability.

Lightweight concrete with a biocomponent and hempcrete exhibit significantly different compressive strength values compared to ordinary concrete. Lightweight concrete, often made with materials like cinder or modified EPS, is designed to reduce the dead load of structures, offering benefits in terms of thermal insulation and environmental impact. However, these advantages often come at the cost of reduced compressive strength. Hempcrete, a sustainable alternative, also prioritizes lightness and thermal properties but similarly suffers from lower mechanical strength compared to traditional concrete. The following sections detail the compressive strength characteristics of these materials.

Lightweight Concrete:

- Lightweight concrete (LWC) typically has a density ranging from 300 to 1850 kg/m^3^, compared to ordinary concrete’s 2200 to 2600 kg/m^3^ [65].
- The compressive strength of LWC can vary significantly based on the materials used. For instance, LWC using the Dreux–Corrise method achieved a compressive strength of 20.59 MPa at 28 days, which is lower than the targeted 24.5 MPa [66].
- Modified EPS and pumice sand in LWC can reduce compressive strength by up to 78.46% compared to normal concrete [67].

Hempcrete:

- Hempcrete, made with hemp hurds and lime, is known for its low density and thermal insulation properties but has a low compressive strength, typically around 9.56 MPa [59].
- The mechanical strength of hempcrete can be influenced by factors such as the granular to binder ratio and compaction during casting [68].

While lightweight concrete and hempcrete offer environmental and structural benefits, their lower compressive strength compared to ordinary concrete can limit their use in load-bearing applications. However, ongoing research into optimizing mix proportions and material properties continues to improve their mechanical performance, potentially expanding their applicability in construction.

### 4.2. Comparative Analysis with Similar Studies on Concrete in Livestock Buildings

Recent literature has shown a significant increase in interest in concrete with biocomponents—both in the form of hempcrete and mixtures with added fibers and plant-derived aggregates or bio-based polymers [48,69,70,71,72,73]. Hempcrete is a well-documented material in residential construction and building renovation, while other bio-modified concretes—based on biopolymers, agro-industrial waste, and biofibers, among others—are studied primarily in the context of infrastructure and structural elements [70,74,75,76]. The reviewed works [48,69,70,71,72,73] did not directly investigate the behavior of these materials in livestock buildings. Therefore, the assessment of suitability for this type of facility must be based on the analysis of general construction research results and knowledge of the specific environmental requirements of animal facilities [48,77,78]. In addition to hempcrete, concretes with the addition of other biocomponents are developing dynamically: plant-based aggregates (rice husks, corn husks, waste wood), bio-based polymer additives, and organic fibers [70,71,72,74,79]. Review studies indicate that so-called bio-based concrete can achieve a wide range of elastic moduli and flexural strength (50–1500 MPa in deflection), with significantly reduced density and increased porosity compared to traditional concrete [74,75]. The goals of these modifications are most often to reduce the carbon footprint, improve thermal insulation properties, and introduce the ability to autogenously seal microcracks (bio-concrete with bacteria) [72,74]. Detailed studies of Slovak hempcrete products have demonstrated significant heat storage capacity [77]. This means that, with appropriate thickness, a hempcrete wall can provide insulation similar to that of lightweight mineral materials while simultaneously exhibiting high thermal inertia, which is important in buildings with natural or mixed ventilation [48,69]. A significant example from the perspective of livestock facilities is the biopolymer polyurethane concrete developed by Murcia et al. [70], where the binder is a bio-based polyurethane with a low carbon footprint [70]. They achieved compressive strength of 20–30 MPa, good resistance to aggressive environments, and very low permeability to water and corrosive solutions, while significantly reducing the carbon footprint compared to Portland concrete [70,72]. In turn, studies on concretes with the addition of wood biomass or rice husks have shown that replacing some of the cement or aggregate with bio-waste lowers density, improves thermal insulation, and can reduce greenhouse gas emissions, although usually at the expense of compressive strength [76,79,80]. Compared to these solutions, hempcrete remains a material with exceptionally low density (250–350 kg/m^3^) and a very good carbon balance, but with low load-bearing capacity (0.2–1 MPa), whereas polymer bio-concretes or mixtures with a small addition of biomass can achieve strengths of several dozen MPa, while only partially retaining their environmental benefits [48,70,75]. From the perspective of livestock buildings, this means that hempcrete is better suited as an insulating filler for the frame, while bio-concretes based on biopolymers or bio-aggregates can perform structural functions in areas exposed to high loads and under intense exposure to corrosive environments [72,74]. In barns, pigsties, and poultry houses, the microclimate is crucial, including the conditions of temperature, relative humidity, air velocity, and concentrations of harmful gases. Climate models of pig facilities (e.g., ThermiPig) show that production results and energy consumption are very sensitive to the parameters of the partitions and ventilation system [78]. Thanks to its moderately low thermal conductivity and high heat capacity, hempcrete can support passive microclimate management strategies by reducing the amplitude of daily temperature and humidity fluctuations [48,77,81]. Bio-concretes with the addition of organic aggregates (rice, wood, or hemp as an additive rather than the main framework) typically have higher thermal conductivity than hempcrete, but still lower than traditional concrete. Their thermal capacity depends primarily on the mineral phase content [76,80]. This means that when used as external walls in livestock buildings, they can improve insulation compared to traditional concrete, but will not necessarily provide the same moisture-buffering properties as hempcrete [48,69]. From the perspective of animal comfort and hygiene, the method of finishing the interior surfaces is of great importance. Hempcrete, when exposed, is an open-pore material, highly “breathable”, but vulnerable to intense moisture and mechanical damage. The use of resin coatings or dense cement mortars leads to reduced vapor permeability and “cutting off” the material from vapor exchange, negating some of its advantages [69]. Bio-concretes based on Portland cement, biopolymers, or hybrid binders generally have lower vapor permeability and lower moisture-buffering capacity, but better resistance to washing and chemical attack [70,75,76]. In livestock facilities, this may mean an advantage for bio-concretes in wet and dirty areas, while hempcrete may be used in areas with milder conditions.

A critical aspect of using bio-based materials in animal housing is their resistance to aggressive environments: manure, urine, ammonia, hydrogen sulfide, and cyclical moisture and drying. In the case of hempcrete, the literature confirms good resistance to mold and fungal growth resulting from the high pH of the lime binder, but there is no systematic study of the effect of slurry and corrosive gases on mechanical properties and porous structure [48,69,73]. It is only indicated that prolonged water saturation significantly increases thermal conductivity, and repeated wetting–drying cycles can lead to shive degradation.

In bio-concrete with the addition of agro-industrial waste (rice husks, plant ash, sawdust), the main problem is increased water absorption and porosity, which, on the one hand, improves insulating properties, but on the other, increases susceptibility to the penetration of aggressive solutions and a decrease in strength over time [76,79,80]. Studies of rice husk concrete show that at high levels of cement substitution with biocomponents, compressive strength can decrease by up to 30–40% compared to the reference concrete [80]. Biopolymer polyurethane concretes, such as biopolymer concrete (BPC), are characterized by very low water permeability, high resistance to corrosive environments, and high compressive strength (20–30 MPa), with a carbon footprint approximately 50% lower than that of Portland cement concrete [70]. While their high polymer content may raise concerns regarding fire temperatures, in terms of chemical resistance and water absorption, they are much better suited for wet areas of livestock buildings than hempcrete or bio-concretes based on plant waste [72]. It is worth noting that some bio-concretes with the addition of biochar and wood aggregates tested in the context of structural concrete demonstrate reduced chloride ion permeability and improved corrosion resistance of reinforcement, which may be important in tanks and manure pits [72,74,76]. However, these studies do not directly address animal housing. A specific requirement for livestock buildings is the need to control gas flow: on the one hand, limiting uncontrolled air infiltration through partitions, and on the other, ensuring the partition’s “breathing” capacity and water vapor diffusion. Research on hempcrete focuses on thermal conductivity, heat capacity, moisture diffusion, moisture-buffering coefficient, and diffusion resistance of partitions, which indirectly indicates water vapor permeability, but not air permeability [48,77,81]. Reviews of hemp-based materials describe porosity, capillary structure, and sorption capacity, but do not provide gas permeability coefficients [69,73]. In the case of bio-concrete with biopolymers and plant waste, water permeability, water absorption, chloride ion diffusion coefficients, and electrical resistance are most often described as measures of structural tightness [70,75,76]. Direct measurement of air (gas) permeability appears sporadically in the context of resistance to chloride penetration. In light of the above, it should be noted that the issue of gas permeability of hempcrete and bio-concrete remains poorly documented. For applications in livestock buildings, where limiting uncontrolled cold air infiltration and enabling the diffusion of water vapor and gases are important, this represents a significant research gap requiring dedicated experimental studies. Comparing the carbon footprint of the analyzed materials, hempcrete stands out for its ability to sequester approximately 300 kg of CO_2_ per m^3^ of material, resulting from the combination of carbon stored in the hemp biomass and the carbonation of the lime binder [69,81]. LCAs of hempcrete partitions indicate significantly lower, and often negative, carbon emissions compared to partitions based on aggregate concrete with mineral insulation or steel panels with a PUR/PIR core [72,73]. Bio-concretes based on replacing part of the cement or aggregate with wood waste, rice husks, biochar, or wood aggregates also reduce greenhouse gas emissions, but the scale of the effect depends on the level of substitution and the energy intensity of waste treatment [76,79,80]. In many cases, emission reductions are achieved compared to the reference concrete, but the negative balance that characterizes hempcrete is not achieved. In the case of biopolymer concrete based on bio-based polyurethane, the carbon footprint is estimated to be approximately 50% lower than in Portland concrete of similar strength, which is due to the high share of components from renewable vegetable oils and the absence of clinker [70]. From the perspective of livestock buildings, the choice between hempcrete and polymer bio-concrete can be understood as a compromise between maximum carbon footprint reduction (hempcrete) and high durability and resistance to aggressive environments (BPC) [72,73].

### 4.3. Scientific Novelty of the Study and Its Innovative Engineering Aspects

The results of the hempcrete (C100) test have important implications for practical engineering applications, especially in the context of structures, where properties such as thermal insulation, moisture resistance, gas permeability and compressive strength are crucial. Here is an extension of the results’ implications with regard to specific engineering applications:

1. Thermal insulation and use in passive buildings.

C100 has a very low thermal conductivity (0.0842 W·(m·K)^−1^), making it an ideal material for applications requiring good thermal insulation. Due to these properties, hempcrete can be used in

- Animal shelters: This material ensures an optimal internal temperature, protecting animals from excessive cold or heat. Additionally, its high insulation helps to reduce energy costs related to heating and cooling.
- Lightweight building panels: Thanks to its thermal insulation properties, C100 is ideal for the production of prefabricated wall panels that will be used in passive construction. This can include both residential houses and public buildings, where energy saving is key.
- Prefabricated wall elements: Due to its ease of forming and good insulation properties, C100 can be used to create lightweight, energy-efficient walls in buildings.

2. Low strength and use in non-load-bearing elements.

C100 has a very low compressive strength (0.36 MPa), which makes it a material more suitable for applications in non-load-bearing elements, such as

- Partition walls: Thanks to its porous structure, C100 can be used to build partition walls that do not carry high loads but provide adequate thermal and acoustic insulation.
- Decorative elements: C100, thanks to its aesthetic and insulating properties, can be used in decorative elements such as panels or architectural ornaments that do not have to bear heavy loads.

3. Moisture absorption capacity and use in moisture-resistant structures.

The high water absorption capacity (99%) of C100 indicates that this material can be used in specific conditions where the accumulation of moisture in the structure is desirable or neutral:

- Animal shelters: Hempcrete can be used in structures that will be in contact with moisture, such as animal housing, which requires a suitable microclimate. It will function well in conditions where moisture is present but does not pose a threat to the building structure.
- Interiors with limited ventilation: C100 can be used in spaces where there is a need for moisture accumulation, such as basements or warehouses, where controlling the humidity level is important.

4. Gas permeability and industrial applications.

Gas permeability, particularly of CO_2_, is another aspect that influences the potential applications of C100. The high resistance of C100 to gas permeability suggests that it can be used in

- Structures requiring minimal CO_2_ permeability: Due to its increased resistance to CO_2_ flow, C100 can be used in industrial buildings that must meet stringent air quality standards or where air pollution is a concern.
- Controlled ventilation: C100 can be useful in structures where maintaining adequate ventilation and air quality parameters is essential, such as laboratories or warehouses storing sensitive products.

5. Acoustic properties and residential applications.

Due to its porous structure, hempcrete can also act as a sound absorbing material. Therefore, this material can be used in

- Apartments and single-family houses: In places requiring acoustic insulation, such as walls between apartments, C100 can provide better sound insulation.
- Public facilities: In conference rooms, theaters or other public spaces where acoustic comfort is important, C100 can help to muffle unwanted sounds.

C100 is a material that has great potential in the field of ecological, insulating and decorative construction, especially where high structural loads are not required. Thanks to its properties, such as low thermal conductivity, high porosity and moisture accumulation, it is more suitable for lightweight structures that focus on thermal, acoustic and moisture protection. It is ideally suited for passive buildings, animal shelters, prefabricated building panels and decorative elements, where its low compressive strength is not a problem.

Another interesting development is the study of agricultural waste in reinforced concrete production [82]. This innovative solution aims to reduce the carbon footprint in construction. Agricultural waste, including coconut shells, rice husk ash, and palm oil ash, represents promising materials due to their mechanical benefits in structural components while reducing CO_2_ emissions. Studies have demonstrated increased compressive strength, tensile strength, and modulus of elasticity. The tested materials have significant potential as sustainable additions to reinforced concrete elements, consistent with global sustainability goals for widespread implementation in the construction industry.

## 5. Conclusions

New scientific information on concrete research:

(1) Hempcrete (C100) is characterized by very good insulating properties—the thermal conductivity coefficient for C100 is 0.0842 W·(m·K)^−1^, which is significantly lower compared to traditional concrete (B1)—3.0480 W·(m·K)^−1^.

The tested C100 class material can be used as an alternative, especially in components that do not bear direct loads, such as infills for external frame walls, partitions, or decorative elements.The compressive strength of the B1 material is 44.60 MPa—this is a typical value for concrete used in structural construction, adapted to transfer external loads. The C100 material obtained a value of 0.36 MPa, and is a material of low strength.

Due to the high content of organic material fragments, hempcrete is characterized by high porosity of the concrete matrix, which translates into huge potential for water accumulation in its structure. The water absorption of sample C100 is 99%, while sample B1, due to its tight structure, achieved a lower value of 2%.

Sample B1 achieved a better value of 0.01 g·(m^2^·s^0.5)−1^, demonstrating high resistance to capillary action, while sample C100 achieved an average value of 0.09 g·(m^2^·s^0.5^)^−1^.

(2) The greatest flow resistance is demonstrated by concrete with the addition of a biocomponent (C100), through which O_2_ or N_2_ flows, while showing significantly lower flow resistance with CO_2_.

A 30% increase in CO_2_ flow resistance was observed for the permeability function of concrete with the addition of a biocomponent (C100) compared to the permeability function of concrete (B1)—a result from this interpretation is that CO_2_ is choked by 25% in terms of the volume flow compared to B1.

The test results for the gas permeability coefficient —using CO_2_ for concrete (B1) and concrete with the addition of a biocomponent (C100)—indicate that the material with the higher gas permeability coefficient is B1, taking into account the geometric mean for the XYZ direction.

The concrete (B1) through which CO_2_ is permeated exhibits the Knudsen diffusion effect—in these conditions, the velocity of particles on the walls of the porous bed is greater than zero; for the gas permeability phenomenon, there is a slip called the Klinkenberg effect.

(3) As a result of the application of two variants of hybrid methods, it should be clearly indicated that concrete with the addition of a biocomponent (C100) is superior—the A2 criterion (thermal conductivity coefficient) and very good insulation of concrete had a decisive influence on this assessment.

The impact of engineering applied to agricultural construction:

(4) The results of the hempcrete (C100) study have significant implications for practical engineering applications:
  - Thermal insulation and use in passive buildings—a very low thermal conductivity coefficient (0.0842 W·(m·K)^−1^), making it an ideal material for applications requiring good thermal insulation;
  - Moisture absorption capacity and use in moisture-resistant structures—high water absorption capacity (99%);
  - Gas permeability and use in industrial applications—particularly CO_2_, is another aspect influencing potential applications;
  - Acoustic properties and use in residential applications—due to its porous structure, hempcrete can also act as a sound-absorbing material.
(5) Limitations of applicability of C100—low strength and use in non-load-bearing elements—very low compressive strength (0.36 MPa) material more suitable for use in non-load-bearing elements; in places requiring sound insulation, such as walls between apartments, can provide better sound insulation.

Outlining the prospects for future research development.

Future research will focus on determining the effect of carbonation on microstructural changes, including pore characteristics and their impact on gas permeability over time. Furthermore, an analysis of the relationship between gas permeability and material durability (including resistance to humidity cycles and microbial growth) is planned, aiming to fill a research gap related to the lack of long-term aging studies and reliable predictive models for the actual operating conditions of hemp composites with bio-additives.

Thermal gas permeability research is also planned—understanding how material density affects porosity and gas transport with temperature. Thermal diffusivity testing will include analysis of the propagation rate of temperature changes within the material. The density–permeability relationship will focus on developing a mathematical model demonstrating how increasing density reduces both thermal conductivity and gas permeability.

Further research directions in the field of DSS:

- Expanding the set of criteria to include life cycle assessment (LCA) and environmental criteria,
- Comparing the results obtained using a larger group of MCDA methods, including hybrid ones, to identify the methods best suited to the analysis of technical issues.

List of Standards

*ISO 12571:2013—Hygrothermal performance of building materials and products—Determination of hygroscopic sorption properties. https://www.iso.org/standard/61388.html (accessed on 20 May 2026).*

Hygrothermal performance of building materials and products—Determination of hygroscopic sorption properties.

This document specifies two alternative methods for determining hygroscopic sorption properties of porous building materials and products:

(a) Using desiccators and weighing cups (desiccator method);
(b) Using a climatic chamber (climatic chamber method).

The desiccator method is the reference method.

This document does not specify the method for sampling.

The methods specified in this document can be used to determine the moisture content of a sample in equilibrium with air at a specific temperature and humidity.

*ISO 8301:1991/Amd 1:2010—Thermal insulation—Determination of steady-state thermal resistance and related properties—Heat flow meter apparatus. https://www.iso.org/standard/52266.html (accessed on 20 May 2026).*

Thermal insulation—Determination of steady-state thermal resistance and related properties—Heat flow meter apparatus.

This international standard is divided into three sections representing the most comprehensive assembly of information required to use the heat flow meter apparatus:

Section 1: General considerations.

Section 2: Apparatus and calibration.

Section 3: Test procedures.

While the user of the method may need to concentrate only on Section 3 for test purposes, he must also be familiar with the other two in order to obtain accurate and precise results. He must be particularly knowledgeable about the general requirements. Section 2 is directed towards the constructor of the apparatus, but he also, in order to build good apparatus, must be familiar with the other sections.

*PN-EN 12390-3—Testing hardened concrete—Part 3: Compressive strength of test specimens. https://www.en-standard.eu/une-en-12390-3-2020-testing-hardened-concrete-part-3-compressive-strength-of-test-specimens/?gad_source=1&gad_campaignid=23589929400&gbraid=0AAAAAD6CNv9cSXoLwaSsRpzylZPPFJSIj&gclid=Cj0KCQjw_b_QBhCSARIsAP6hR4eeROhbsFjFoD8IxNc3PqrH7803Y6pmJ0altrqjqTVOPKbCWVbf3xQaAqk9EALw_wcB (accessed on 20 May 2026).*

Testing hardened concrete—Part 3: Compressive strength of test specimens.

This document provides a method for determining the compressive strength of concrete samples.

*PN-88/B-06250—Characteristics and Requirements of Ordinary Concrete. https://www.studocu.com/pl/document/politechnika-rzeszowska-im-ignacego-lukasiewicza/mechanika-budowli/pn-88-b-06250-beton-zwykly-norma-archiwalna/56544263 (accessed on 20 May 2026).*

Normal concrete.

The standard covers requirements for the properties of components and the properties and testing of concrete mixes and normal concrete.

*PN-EN 772-11—Thermal and moisture tests of building materials. https://wbiis.zut.edu.pl/dla-przemyslu/badanie-materialow-budowlanych.html (accessed on 20 May 2026).*

Test methods for masonry units—Part 11.

This standard specifies procedures for determining water absorption due to capillary action and initial water absorption. It applies to concrete masonry units (ACM), artificial and natural stone. The standard helps determine how quickly a material absorbs water.

## Figures and Tables

**Table 1:** Thermal conductivity of selected concretes [own study].

Type of Concrete	Thermal Conductivity λ, W(m·K)^−1^
Hempcrete	0.05–0.10
Traditional concrete	1.4–3.2

**Table 2:** Material composition of the samples used for testing [own study].

Designations	Composition in Percentage	Aggregate [g] Fraction 0–2/2–8/8–16	Hemp Shive [g]	Cement CEM I 42,5 R [g]	Fluidized Fly Ash (FPL) [g]	Water [g]
C100 (CS1)	C59-P29-A12	0	1000	2000	400	2500
B1	C16-K84	734/550/550	0	350	0	175

Legend: C—cement CEM I 42.5 R; P—hemp shive; A—fly ash; K—stone aggregate.

**Table 3:** Results for the thermal conductivity coefficient (λ) of the tested materials: concrete (B1), concrete with biocomponent (C100) (own study).

Research Material	Designations	Composition %	Thermal Conductivity Coefficient λ, W·(m·K)^−1^
concrete with biocomponent	C100 (CS1)	C59-P29-A12	0.0869
concrete	B1	C16-K84	3.0480

Legend: C—cement CEM I 42.5 R; P—hemp shive; A—fly ash; K—stone aggregate.

**Table 4:** Compressive strength test results for the tested materials: concrete (B1), concrete with biocomponent (C100) (own study).

Research Material	Designations	Composition %	Compressive Strength MPa
concrete with biocomponent	C100 (CS1)	C59-P29-A12	0.36
concrete	B1	C16-K84	44.60

Legend: C—cement CEM I 42.5 R; P—hemp shive; A—fly ash; K—stone aggregate.

**Table 5:** Water absorption test results for the tested materials: concrete (B1), concrete with biocomponent (C100) (own study).

Research Material	Designations	Composition %	Water Absorption, %
concrete with biocomponent	C100 (CS1)	C59-P29-A12	99
concrete	B1	C16-K84	2

Legend: C—cement CEM I 42.5 R; P—hemp shive; A—fly ash; K—stone aggregate.

**Table 6:** Capillary rise test results for the tested materials: concrete (B1), concrete with biocomponent (C100) (own study).

Research Material	Designations	Composition %	Water Absorption, %
concrete with biocomponent	C100 (CS1)	C59-P29-A12	99
concrete	B1	C16-K84	2

Legend: C—cement CEM I 42.5 R; P—hemp shive; A—fly ash; K—stone aggregate.

**Table 7:** List of technical parameters constituting evaluation criteria (prepared by: D. Fabianowski).

Variants	A1 (D)	A2 (D)	A3 (S)	A4 (D)	A5 (D)	A6* (D)	A7 (D)
	Density [kg/m^3^]	Thermal Conductivity Coefficient λ [W/(mK)]	Compressive Strength [MPa]	Absorbability [%]	Capillary Absorption [g/(m^2^s^0.5^)	Gas Permeability 10^−7^ m^2^	Cost 1 m^3^·$^−1^
B1	2184	3.05	44.6	2	0.01	1.73	102.54
C100	486	0.08	0.36	99	0.90	1.57	117.81
ID	250	0.03	50	1	0.01	1.0	50
AID	3000	3.5	1	100	1.0	3.0	250

Designations: B1—reference concrete, C100—concrete with biocomponent, ID—ideal variant, AID—anti-ideal variant, A6—average value from gas permeability tests for oxygen, nitrogen and carbon dioxide (from flow resistance measurements for 6 MPa), S, D—stimulant and destimulant, respectively.

**Table 8:** The values of the weights of individual criteria and the results of normalization of diagnostic variables using the UTI method, together with the evaluation ranking vectors (prepared by: D. Fabianowski).

Variants	Criteria							AHP + Hellwig’s Method	AHP + TOPSIS
	A1 (0.030)	A2 (0.414)	A3 (0.054)	A4 (0.092)	A5 (0.072)	A6 (0.206)	A7 (0.132)		
B1	0.297	0.130	0.892	0.990	1.000	0.635	0.737	0.59	0.37
C100	0.914	0.986	0.005	0.010	0.101	0.715	0.661	0.67	0.74

**Table 9:** Comparative analysis of key properties of ordinary concrete (B1), lightweight concrete with biocomponent (C100) and hempcrete (prepared by K. Borek).

Property	Ordinary Concrete (B1)	Lightweight Concrete (C100)	Hempcrete [61,63,64]
Thermal Conductivity	1.0 to 2.0 W/m·K	0.3 to 0.8 W/m·K	0.04 to 0.1 W/m·K
Compressive Strength	20 to 40 MPa	5 to 15 MPa	0.5 to 2.0 MPa
Water Absorption	1% to 3% by weight	5% to 10% by weight	10% to 20% by weight
Gas Permeability	10^−19^ to 10^−17^ m^2^	10^−17^ to 10^−15^ m^2^	10^−15^ to 10^−13^ m^2^

## Data Availability

The original contributions presented in this study are included in the article. Further inquiries can be directed to the corresponding author.
